# Development and validation of a food group system for intake control in people with diabetes: SMARTCLOTH-Database

**DOI:** 10.1371/journal.pdig.0001498

**Published:** 2026-07-09

**Authors:** María García-Rodríguez, Fernando León-García, Rafael Molina-Luque, María Pilar Villena-Esponera, Manuel Romero-Saldaña, Guillermo Molina-Recio

**Affiliations:** 1 Faculty of Health Sciences, UTAMED (Universidad Tecnológica Mediterráneo), Málaga, Spain; 2 Department of Electronic and Computer Engineering, University of Córdoba, Córdoba, Spain; 3 Lifestyles, Innovation and Health research group (GA-16). Maimonides Biomedical Research Institute of Cordoba (IMIBIC), Córdoba, Spain; 4 Advanced Informatics Research Group (GIIA), TIC-252. University of Córdoba, Córdoba, Spain; 5 Nursing, Pharmacology and Physiotherapy Department, University of Córdoba, Córdoba, Spain; 6 Faculty of Arts and Social Sciences, International University of La Rioja, Logroño, Spain; Yonsei University College of Medicine, KOREA, REPUBLIC OF

## Abstract

This study presents the development and validation of the SMARTCLOTH-Database, a structured food group system designed to support dietary management for individuals with diabetes. The database organises foods into 20 nutritionally coherent groups, distinguishing between raw and cooked forms, based on Spanish dietary patterns and statistical criteria. Nutritional values were calculated using weighted averages derived from national food consumption data and multiple international food composition databases. To validate the system, a computational tool was developed to generate daily menus automatically and compare nutritional outputs against a gold standard reference (BDCA). The comparison showed small mean differences (less than ±22 kcal, < 2 g carbohydrates, < 0.6 g protein, and <1.5 g lipids), with high concordance demonstrated through intraclass correlation coefficients (ICC ≥ 0.75) for carbohydrates and proteins across most analyses. These results indicate that the SMARTCLOTHDatabase provides accurate and reliable estimates of macronutrient content, making it suitable for integration into digital tools aimed at improving dietary self-management in people with diabetes.

## Author summary

People with diabetes often need to estimate the nutritional content of meals, especially carbohydrates, to support daily decisions about diet and treatment. However, this task often relies on food composition tables, exchange lists, or broad food group classifications, which can be difficult to use and may not reflect the diversity of real meals. In this study, we developed and validated the SMARTCLOTH-Database. This new system organises foods into 20 nutritionally coherent groups with scientifically calculated average values for energy, carbohydrates, proteins, and lipids. Compared with the traditional six-group classification commonly used in dietary education for people with type 1 diabetes, this system offers a more detailed and flexible approach. Our validation showed that the database provides reliable estimates, particularly for carbohydrates and proteins, supporting its scientific validity for dietary monitoring. The SMARTCLOTH-Database will be integrated into the SMARTCLOTH device, a hardware system currently under development that combines food weighing with nutritional information from these validated groups. This resource will allow users to know, in real time and with scientific support, the nutritional value of the foods served on a plate.

## 1. Introduction

### 1.1. Databases on food composition and food groups

Diabetes mellitus is one of the most prevalent chronic diseases today and is associated with a significant health, economic and social impact worldwide [1]. In the design of nutritional interventions for people with diabetes, it is necessary to utilise food composition databases (FCDBs) to obtain standardised and validated information on the macronutrient and micronutrient content of foods. The SMARTCLOTH project and its database are designed to support nutritional management in both Type 1 and Type 2 diabetes, where dietary regulation plays a central role in metabolic control.

The most widely used and internationally recognised FCDBs include the USDA FoodData Central [2], the international network of food data systems INFOODS [3], and the Spanish Table of Food Composition (BEDCA) in the Spanish-speaking context [4]. Its structure facilitates the classification of foods into groups based on nutritional, technological, or functional similarities, which is crucial for designing therapeutic diets. These food composition databases and classification systems serve as essential tools for implementing effective dietary monitoring strategies in clinical practice.

### 1.2. Monitoring dietary intake in people with diabetes

Dietary control, understood as the structured and quantitative management of macronutrient intake—particularly carbohydrates—to optimize glycaemic response and medication adjustment is a fundamental pillar in the comprehensive management of type 1 (DM1) and type 2 (DM2) diabetes mellitus, not only because of its direct impact on glycemic control, but also because it influences quality of life, patient perception, and the prevention of long-term complications [5]. Several studies have shown that proper dietary planning, prioritising carbohydrate quality and quantity, significantly improves glycosylated haemoglobin (HbA1c) levels, without adverse effects on body weight or insulin requirement, especially in patients with DM1 who use carbohydrate counting as a therapeutic tool [5]. In patients with type 2 diabetes mellitus (DM2), a balanced diet rich in fibre and low in simple sugars and saturated fats helps improve insulin sensitivity and lipid profile, key elements in the prevention of cardiovascular events and other associated complications [6,7].

Dietary management also has a synergistic relationship with pharmacological treatment. Recent studies have shown that appropriate nutritional intervention can enhance the efficacy of hypoglycaemic drugs, allowing a reduction in the doses required or even the suspension of some drugs in patients with reasonable metabolic control [8]. Nutritional therapy, administered by trained professionals, has been shown to be comparable in efficacy to some pharmacological therapies for reducing HbA1 [9]. For example, dietary interventions based on a Mediterranean or low glycaemic index diet, applied in conjunction with treatments such as metformin or SGLT2 inhibitors, have been shown to significantly improve metabolic control and reduce disease progression [10].

A structured and personalized diet is associated with better perceived disease control, increased motivation to adhere to treatment, and better overall quality of life [11]. Patients who receive ongoing nutrition education have a lower incidence of chronic complications such as retinopathy or diabetic nephropathy, and report greater satisfaction with their treatment [12]. In fact, the empowerment that comes from knowledge and self-regulation of dietary intake results in lower levels of anxiety, better glycaemic control and greater autonomy [11,13].

These approaches improve glycaemic control and dietary flexibility by enabling individuals to adjust insulin or hypoglycaemic medication based on actual carbohydrate intake [7–10]. Programmes such as DAFNE and the “Spanish *Conteo de Raciones* (Portion Counting) model” have consistently demonstrated reductions in HbA1c and improvements in quality of life [11–13].

Despite their clinical efficacy, long-term adherence remains limited, particularly among younger individuals or those with lower nutritional training [14,15]. The success of these interventions largely depends on patients’ ability to accurately estimate food composition and portion sizes, which requires continuous education, cognitive effort, and often professional support. This underscores the need for complementary digital tools that simplify calculations, enhance patient autonomy, and promote the continuity of nutritional self-management. Several structured approaches have been developed to facilitate these calculations and support dietary self-management in clinical practice.

### 1.3. Food groups, exchange diets and portion control in people with diabetes

Among the most commonly used approaches for monitoring carbohydrate intake are carbohydrate counting (CC), highlighting the exchange method and the portion method (*Clinic*) in Spain. CC, particularly useful in patients with DM1 receiving intensive insulin therapy, allows for the adjustment of the rapid insulin dose according to the carbohydrate content of meals, which has been shown to improve glycaemic control and quality of life [5,14]. However, its effectiveness is highly dependent on the patient’s ability to accurately estimate carbohydrate content, a task that remains challenging and frequently subject to error [14].

Exchange and portion methods offer a more structured and accessible alternative for certain patients, especially in contexts of low nutritional literacy. These methods group foods with similar compositions, allowing for substitutions within each group. Although they facilitate meal planning, their usefulness is limited by the need for cultural adaptations, lack of achieving a very high precision estimates and limited meal variety that may affect adherence [15,16]. Additionally, it has been noted that the number of food groups included in these methods may be insufficient to reflect the dietary diversity of many populations accurately. In particular, lists of traditional exchanges often do not include foods typical of particular cultures or local preparations, which makes their practical application difficult. This limitation can lead to poor adherence, errors in nutrient estimation and a perception of rigidity that compromises the effectiveness of the nutritional plan [15]. Therefore, several authors propose updating and adapting these systems to the specific dietary patterns of each region to improve their clinical utility and patient acceptance [15].

Although internationally recognised food composition databases such as BEDCA, USDA or INFOODS are available, most were designed for descriptive or epidemiological purposes rather than for integration into automated calculation systems or interactive dietary monitoring tools. Furthermore, their structure does not include consumption-weighted data or groupings based on clinical and nutritional criteria specific to diseases such as diabetes mellitus. This limitation restricts their direct applicability in technological tools that require fast, standardised, and culturally representative estimations. Several authors have highlighted that traditional exchange systems and composition tables do not adequately reflect regional food diversity, which can lead to clinically relevant errors in macronutrient estimation and negatively affect dietary adherence [14–16]. In this context, the development of a food group–structured and consumption-weighted database such as SMARTCLOTH-Database addresses not only a technical need within the project but also a scientific and clinical demand for more accurate, interoperable, and culturally contextualised food classification systems.

Given these limitations in traditional dietary management approaches and the growing need for more accessible and accurate tools, digital technologies have emerged as promising solutions to bridge the gap between clinical recommendations and patient self-management. In the last decade, technological solutions have been developed to support the estimation of carbohydrate content and improve patient self-management. Mobile applications, such as Carbulin, allow not only advanced carbohydrate counting but also the calculation of personalised insulin doses and nutrition education tailored to the user’s cultural context [13]. These tools reduce estimation errors and facilitate decision-making, but their effectiveness remains dependent on digital literacy and patient engagement with their use [14,17].

Taken together, these approaches show considerable potential for improving the nutritional management of diabetes. However, their methodological limitations, especially in terms of counting accuracy, individual adaptation, and reliance on manual data entry, justify the need for new, integrated, technological and personalised strategies.

### 1.4. The SMARTCLOTH project

SMARTCLOTH is an interactive smart tablecloth designed to improve dietary adherence in people with diabetes through real-time nutritional monitoring (https://smartcloth.org/) [18]. The device automatically calculates energy and macronutrient content as food is served on the plate, eliminating the need for manual data entry and reducing estimation errors inherent to traditional carbohydrate counting methods. To enable accurate and culturally appropriate nutritional calculations, SMARTCLOTH requires a specialized food database that combines clinical relevance with real-world consumption patterns. This need motivated the development of SMARTCLOTH-Database, which serves as the nutritional core of the system and is the focus of validation in the present study.

### 1.5. Objective

This study aims to address the limitations of current food classification systems through the development and validation of SMARTCLOTH-Database. Specifically, this database provides a consumption-weighted, culturally adapted food grouping system designed to enable accurate automated dietary monitoring in people with diabetes, reducing estimation errors and eliminating the need for manual data entry inherent to traditional carbohydrate counting methods. This structured approach is essential for the SMARTCLOTH device to function seamlessly and provide users with a simplified, user-friendly dietary self-management experience.

## 2. Materials and methods

### 2.1. Initial choice of food groups

For the development of the SMARTCLOTH-Database, we initially opted for a food classification based on the food group exchange lists widely used by clinical nutritionists in Spain for dietary planning [19,20], and differentiated, when necessary, between raw and cooked foods. This approach has significant advantages over the classical division into six groups in portion diet (dairy, farinaceous, fruits, vegetables, meat/fish/eggs and fats), commonly used in care settings under the so-called *Clínic method* [21,22]. Although this traditional classification facilitates patient understanding, its simplicity prevents capturing the nutritional complexity and heterogeneity within each group, which limits its usefulness when precision is required in nutrient calculations or the design of interactive devices such as SMARTCLOTH.

For this reason, the model proposed by Marques-Lopes et al. was adopted as a reference, which structures the food groups based on objective statistical criteria applied to key macronutrients (energy, carbohydrates, proteins and fats), allowing the creation of more adjusted and representative interchangeable rations of the Spanish dietary pattern [19]. This structure was later extended in a second paper introducing additional variables such as fibre, simple sugars, cholesterol or specific fatty acids, thus facilitating its application to more complex clinical situations This more detailed and nutritionally sound classification serves as the basis for constructing the SMARTCLOTH-Database (S1 Table).

### 2.2. Weighing the nutritional value of the food groups and the definition of the Gold Standard for validation

The SMARTCLOTH-Database was constructed based on an extended classification of 20 food groups, encompassing a total of 199 types of food, which differentiate between raw and cooked. The criteria used for this classification are based on the clinical and dietary relevance of each food in the nutritional management of diabetes, considering the predominant nutritional contributions of each food and its frequency of consumption in the Spanish population [23].

For each of the food groups, a weighted average value was calculated for energy (kcal) and its macronutrient composition (carbohydrates, proteins, lipids) in grams per 100 grams of food.

For the weighing, the historical series of food consumption in Spanish households for 2023, published by the Ministry of Agriculture, Fisheries and Food of the Spanish Government [23], was used. The steps followed were as follows: i) the prevalence of consumption was determined for each food in each group, according to the data published by the Ministry (2023); ii) from the prevalence of consumption, each food within each group was weighed; iii) with the established weighing, the average nutritional values of each food group were calculated.

To calculate the primary nutritional value of each food group (before weighing), data from various national and international food composition databases were used, selecting in each case the source that best represented the actual nutritional profile of each food. The databases used included the Spanish Table of Food Composition (BEDCA) [4], the USDA FoodData Central [2] and other sources included in the international INFOODS network [3]. This strategy enabled the obtaining of more representative and adapted nutritional values, considering both the local availability of the feed and its specific composition. The data thus selected were generically named FCDB (for its acronym in Spanish, “*Base de datos de Composición de Alimentos*”).

In relation to commercial or branded products, the SMARTCLOTH-Database did not include them as individual items. Instead, representativeness was ensured by using the official food consumption data published by the Spanish Ministry of Agriculture, Fisheries and Food (MAPA, 2023) [23], which already account for the market distribution and frequency of branded items in the national diet.

Nutrient composition values were taken from official food composition tables (BEDCA [4], USDA [2], and FAO/INFOODS [3]), which provide standardized analytical data rather than label-derived values. This approach was adopted to ensure methodological consistency and analytical reliability, as nutrient values declared on commercial labels may vary due to formulation changes, rounding, or differing analytical methods. The use of official databases therefore provides more stable and comparable reference values for nutritional modelling.

**Fig 1:**
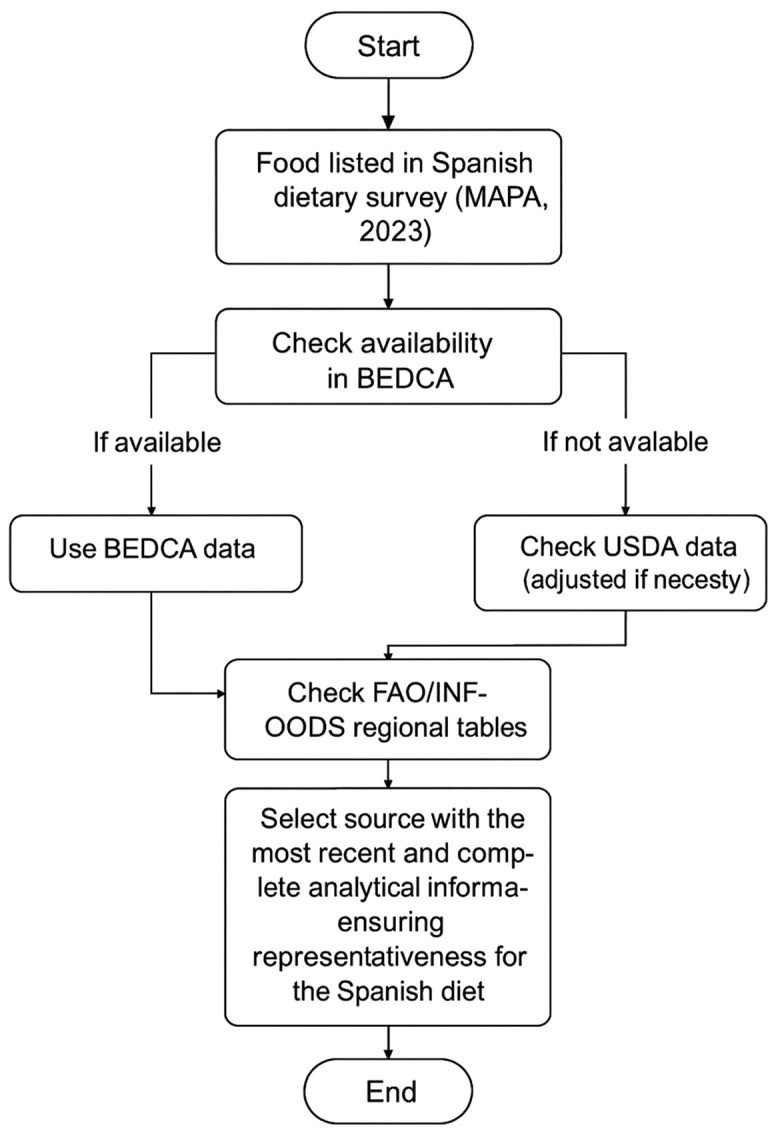
Decision flow for the selection of nutrient composition data sources. Hierarchical procedure used to select the most appropriate food composition database (BEDCA, USDA Food Data Central, or FAO/INFOODS) for each food item based on data availability, completeness, and regional representativeness.

To address the variability that can arise between different food composition databases, a hierarchical selection procedure was established to determine which source to use for each item. The BEDCA database [4] was adopted as the primary reference, given its official nature and representativeness for the Spanish population. When a food was not available or its composition data were incomplete in BEDCA, information was retrieved from USDA FoodData Central [2] or, when appropriate, from FAO/INFOODS [3] regional databases, prioritizing those records that best reflected the food’s origin and typical preparation in Spain. In cases where several options were available, the source providing the most recent and complete analytical information was selected to ensure internal consistency and comparability across foods (Fig 1) (S1 Table).

### 2.3. Development of the validation software

As part of the SMARTCLOTH model validation process, software was developed to automatically generate daily diets based on structured rules. These rules specify the food groups for each meal (breakfast, lunch, dinner, and snack), along with portion adjustments and selection probabilities for random variations.

Each generated daily menu comprises specific foods and their quantities in grams for each meal. The nutritional content is then evaluated using both FCDB (constructed from multiple food composition databases [2–4]) and SMARTCLOTH (the validation target). Both models estimate energy (kcal), carbohydrates (g), lipids (g), and proteins (g). The tool organizes results from both models at the following aggregation levels for analysis:

(i) Aggregation by food item: Nutritional information is obtained for each food item, based on the quantity in grams included in each dish of each daily menu. This food item is the most basic level of information on which the following levels of aggregation are built.
(ii) Aggregation by dish: Nutritional information for food items is aggregated by dish (breakfast, lunch, dinner or snack).
(iii) Aggregation by food group: Foods are aggregated into the groups proposed by the SMARTCLOTH model (whole dairy products, legumes, etc.).
(iv) Aggregation by day: The information is aggregated for the entire day, regardless of which food is consumed in which dish.

The analysis uses nutritional information from both models for daily menus over a defined period. Differences between SMARTCLOTH and FCDB are quantified and statistically analyzed at four levels: food, group, meal, and day. Descriptive statistics (mean, maximum, minimum, and standard deviation) are calculated for each level across the entire period. This approach validates the overall consistency of the SMARTCLOTH model, identifies systematic deviations and patterns of overestimation or underestimation, and evaluates the accuracy of nutrient estimates at different aggregation scales.

Additionally, to ensure realistic nutrient estimation for cooked dishes, the system incorporated nutrient adjustment coefficients derived from the Nutriplato Software [24]. This tool applies standardised thermal processing retention factors to account for nutrient losses and compositional changes during cooking, allowing for more accurate macronutrient and energy estimation in prepared meals.

**Fig 2:**
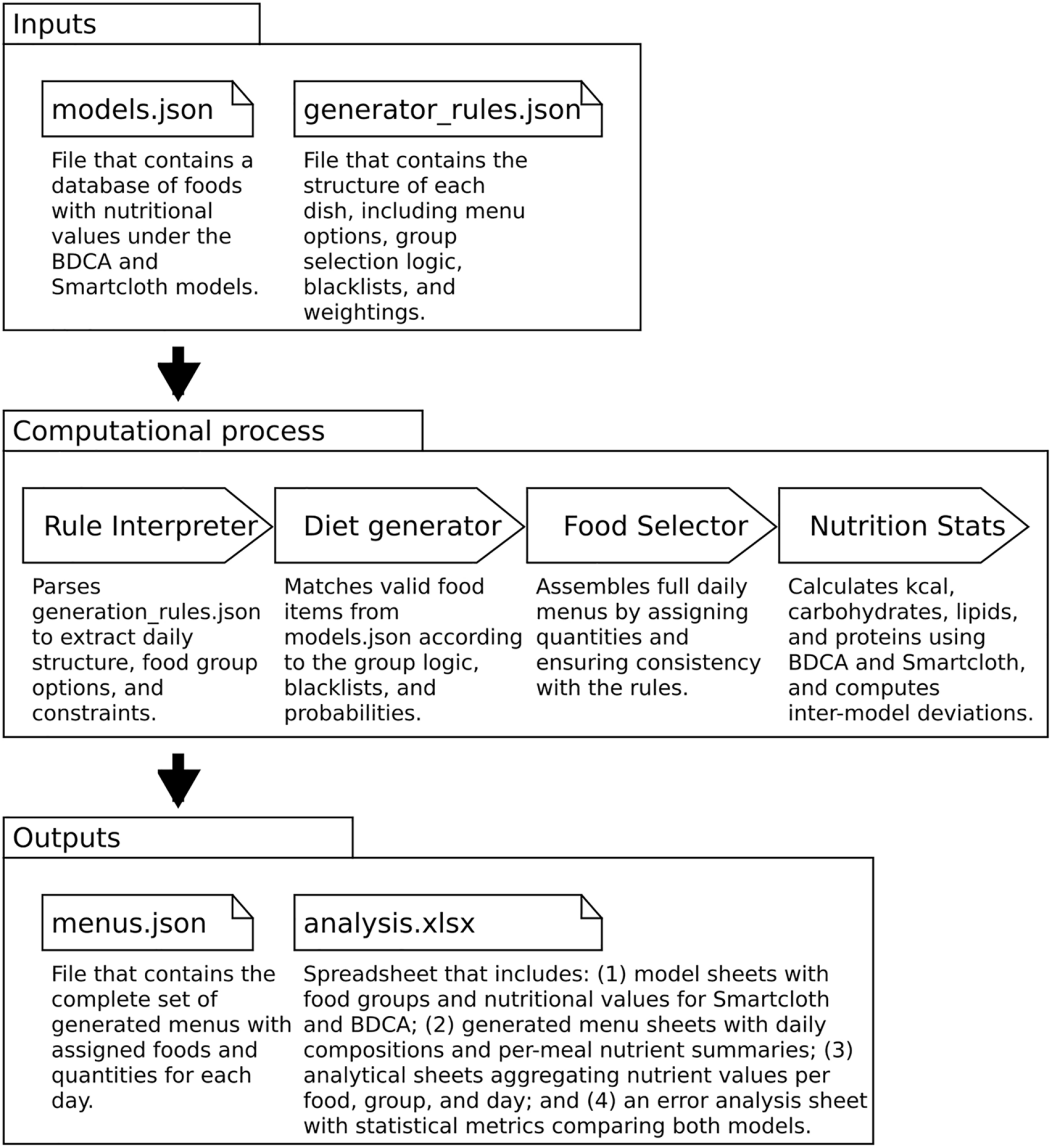
General diagram of the generation process.

Fig 2 illustrates the automatic menu generation process. The system uses two main input files: one with FCDB and SMARTCLOTH model data (foods, groups, and nutritional values) and another with rules specifying how to generate daily meals from SMARTCLOTH groups. The software then selects foods, assigns portions, and generates complete menus for the specified period. Results are stored in two output files: a structured JSON file with the generated menus and an Excel file (.XLS) with nutritional calculations and statistical analyses at the described levels. Further technical details of the software are provided in **S1 Appendix**, and the resulting spreadsheet used for model validation is available in S1 Data.

### 2.4. Validation strategy and statistical analysis

The external reliability of the SMARTCLOTH-Database was determined by analysing the criterion validity, comparing nutritional results with FCDB, which was selected as the gold standard. The degree of clinical agreement between the two methods was calculated using the intraclass correlation coefficient (ICC) for both single measurements and average measurements, for each outcome variable (nutritional value). The Intraclass Correlation Coefficient (ICC) was estimated using a two-factor mixed-effects model with an absolute agreement approach, considering subjects as random effects and assessment methods as fixed effects. This model assesses the degree of agreement between measurements made by a specific set of raters, taking into account both intersubject variability and possible systematic differences between raters. In addition, the ICC has been determined based on average measurements because the objective is to determine the reliability of the measurements between the two instruments.

The ICC model used corresponds to ICC (3,k) for average measures, where the “3” indicates a two-way mixed-effects model and “k” represents the number of measurements averaged. This model was selected because it is appropriate when assessing agreement between a fixed set of methods (SMARTCLOTH-Database and FCDB) applied to multiple subjects, and when the goal is to evaluate the reliability of mean scores rather than individual ratings. The ICC analysis was conducted independently at three levels of aggregation: individual food items, composite dishes, and complete daily menus. The absolute agreement definition was chosen over consistency to account for both systematic bias and random error. ICC values were calculated with 95% confidence intervals. Internationally accepted criteria were followed for the interpretation of the ICC values [25]: Poor (<0.40), Sufficient (0.40-0.59), Good (0.60-0.74) and Excellent (0.75-1) [26]. Additionally, the mean and standard deviation of the mean differences were calculated for each nutritional value obtained by both methods. To analyse the assumption of normality of the data, the Kolmogorov-Smirnov test with the Lilliefors correction and the P-P and Q-Q graphs were used.The statistical programme JAMOVI ver. 2.3.28 was used.

### 2.5. Reproducibility and data availability

To ensure the reproducibility of the results and the methodology used in this study, a comprehensive step-by-step technical protocol has been deposited in protocols.io [27]. This protocol includes the complete workflow for data generation using the Python-based validation software, the configuration of the virtual environments, and the specific parameters for the statistical assessment conducted in JAMOVI. The repository containing the source code and the validation datasets is also linked within the aforementioned protocol to allow for full independent verification of the SMARTCLOTH-Database.

## 3. Results

### 3.1. Number of groups, nutritional values and foods contained in each one, differentiating raw and cooked

SMARTCLOTH-Database includes a total of 20 food groups, selected for their clinical and dietary relevance in the management of diabetes. Of these, six groups have differentiated versions according to how they are consumed: raw or cooked, since heat treatment significantly alters their energy density and macronutrient composition. This distinction affects foods such as vegetables, legumes, meat, fish, and eggs, and has been incorporated to improve the accuracy of nutritional calculations within the SMARTCLOTH system.

Table 1 presents the average nutritional values per 100 grams or 100 millilitres of food in each group and subgroup, including energy (kcal), carbohydrates (Carb), lipids (Lip), and protein (Prot). This information served as the basis for the system simulations, allowing rapid estimation of the nutritional composition of complete daily meals and menus.

**Table 1:** Nutritional composition of the food groups in the SMARTCLOTH-Database.

Food group	Subgroup	kcal	Carb	Lip	Prot
Whole milk	Raw	69.584	4.831	4.156	3.576
Semi-skimmed dairy	Raw	47.29	4.95	1.74	3.32
Skimmed milk products	Raw	33.93	4.78	0.28	3.38
Sweetened dairy products	Raw	85.98	13.16	2.57	3.01
Dairy desserts	Raw	184.84	24.78	8.06	3.56
Sugar and sweets	Raw	351.62	84.46	2.47	2.62
Vegetables	Raw	24.54	4.12	0.37	1.48
Vegetables	Cooked	22.82	2.35	0.37	1.58
Fresh fruit, dried fruit and juices	Raw	48.055	11.196	0.2497	0.801
Cereals and tubers	Raw	210.53	44.52	1.42	6.22
Cereals and tubers	Cooked	96.17	23.75	0.51	1.95
Legumes	Raw	322.36	55.23	3.27	21.48
Legumes	Cooked	117.62	18.68	1.46	8.58
Confectionery, pastry and other	Raw	389.462	50.55	14.204	7.278
A. Very lean protein	Raw	99.47	1.01	1.76	19.3
A. Very lean protein	Cooked	94.5	0.13	1.31	19.78
Lean protein foods	Raw	138.956	0.443	7.046	18.477
Lean protein foods	Cooked	173.116	0.737	8.378	22.879
A. Semi-fatty protein	Cooked	147.69	0.64	10.67	12.98
A. Semi-fatty protein	Crude	155.25	0.64	11.26	13.47
A. Fatty protein	Raw	279.04	0	21.6	20.61
A. Fatty protein	Cooked	281.56	0	22.6	18.69
A. Very fatty protein	Raw	319.11	3.507	28.48	13.348
Foods rich in MUFA	Crude	728.325	3.43	78.811	2.89
SFA-rich Foods	Raw	561.78	1.78	61.15	1.26
PUFA-rich Foods	Crude	846.23	2.487	93.285	2.487
Other fat blends	Crude	826.86	0.07	91.36	1.07

*MUFA: Monounsaturated fatty acids*

*SFA: Saturated fatty acids*

*PUFA: Polyunsaturated fatty acids*

### 3.2. Comparisons of energy and macro (CH, LIP, PROT) distribution by food groups, meals, daily menus and totals. Tables with mean, maximum and minimum differences

Through the computational tool described above, and by combining the 199 meals from the SMARTCLOTH-Database according to nutritional rules, a total of 365 menus were generated. Each menu, in turn, consisted of three dishes (one breakfast, one lunch and one dinner).

Table 2 presents the differential analysis between the SMARTCLOTLOTH-Database and FCDB (reference), broken down by dishes and the food used to configure these dishes, indicating the mean (standard deviation) and the range (minimum-maximum).

**Table 2:** Differential analysis by dishes and food between SMARTCLOTH-Database and FCDB (reference) for the main nutritional values.

ANALYSIS	Calories (kcal) Mean (SD) Range (min., max.)	Carbohydrate Mean (SD) Range (min, max)	Protein Mean (SD) Range (min, max)	Lipids Mean (SD) Range (min, max)
**BREAKFAST**				
**FOOD** **n = 1460**	10.9 (48.8) 300.1 (-83.2, 216.9)	-0.27 (3) 28.4 (-17, 11.4)	-0.1 (1.3) 10.6 (-9.4, 1.2)	1.4 (5.5) 29.6 (-5, 24.6)
**DISHES** **n = 365**	43.6 (87.4) 346 (-10.2, 244)	-1.1 (5.8) 33.5 (-22.8, 10.7)	-0.4 (2.4) 13.9 (11.6, 2.3)	5.6 (9.6) 30 (-5, 25)
**LUNCH**				
**FOOD** **n = 2008**	4.7 (42.9) 348 (-134.7, 213.6)	-0.11 (0.56) 40.5 (-29.8, 10.6)	0.1 (2.2) 36.4 (-11.3, 25.1)	0.36 (4.3) 41.3 (-17.1, 24.2)
**DISHES** **n = 365**	25.7 (96.9) 528 (-234.8, 293.8)	-0.63 (13.3) 62.3 (-43.1, 19.2)	0.54 (5.1) 39.2 (-14.4, 24.8)	2 (9.7) 50.4 (-19.8, 30.6)
**DINNER**				
**FOOD** **n = 1654**	4.7 (46.7) 347 (-128.5, 218.4)	0.33 (3.9) 36.9 (-25.9, 11)	0.12 (2.4) 40.4 (-11.9, 28.5)	0.22 (4.6) 40.8 (-16.1, 24.7)
**DISHES** **n = 365**	21.2 (91.4) 588 (-239.4, 349.1)	1.5 (8.4) 39.4 (-26.6, 12.8)	0.54 (5.1) 42.7 (-12.7, 30)	0.98 (9.6) 48.5 (-20.5, 28)

As can be seen, the mean values of the differences between SMARTCLOTH-Database and FCDB for the nutritional parameters are less than 26 kcal, less than 2 g for CH, a maximum of 0.54 for protein, and less than 1.5 g for lipids. In terms of ranges, the highest values are obtained in the elaborated dishes, with 588 kcal at dinner, 62.3 g of CH at lunch, 42.7 g of Protein (dinner), and 50.4 g of lipids (lunch).

### 3.3. Statistical analysis of concordance for groups, meals, menus and totals

Additionally, criterion validity was determined by analysing the clinical agreement between SMARTCLOTH-Database and FCDB (Table 3), by calculating the intraclass correlation coefficient for single measures (ICCs) and average measures (ICCa). Clinical agreement for carbohydrate, the essential macronutrient for diabetes mellitus control, was excellent (CCI>= 0.75) for all comparisons except for the agreement between dinner dishes (ICCs = 0.691).

**Table 3:** Clinical concordance between SMARTCLOTH-Database and BEDCA (reference) for main nutritional values. ICC average measures (ICCa).

ICC	Calories (kcal)	Carbohydrates	Proteins	Lipids
**BREAKFAST**				
**FOOD (n = 1460)**				
**ICCa**	0.59 (0.54-0.64)	0.95 (0.95-0.96)	0.982 (0.98-0.984)	0.746 (0.7-0.78)
**DISHES (n = 365)**				
**ICCa**	0.414 (0.21-0.56)	0.93 (0.91-0.94)	0.963 (0.95-0.97)	0.17 (-0.12 -0.32)
**LUNCH**				
**FOOD (n = 2008)**				
**ICCa**	0.867 (0.85-0.88)	0.95 (0.94-0.96)	0.977 (0.97-0.98)	0.856 (0.84-0.87)
**DISHES (n = 365)**				
**ICCa**	0.849 (0.8-0.88)	0.88 (0.85-0.9)	0.949 (0.94-0.96)	0.662 (0.58-0.73)
**DINNER**				
**FOODS (n = 1654)**				
**ICCa**	0.86 (0.85-0.87)	0.96 (0.95-0.96)	0.974 (0.97-0.99)	0.847 (0.83-0.86)
**DISHES (n = 365)**				
**ICCa**	0.75 (0.69-0.8)	0.817 (0.77-0.85)	0.928 (0.91-0.94)	0.622 (0.65-0.69)

For the remaining nutritional variables, protein achieved the highest clinical agreement between the two methods, excellent in all comparisons (ICC >=0.75). In contrast, the concordance for lipids had the lowest ICC results, with the analysis of breakfast dishes standing out.

**Fig 3:**
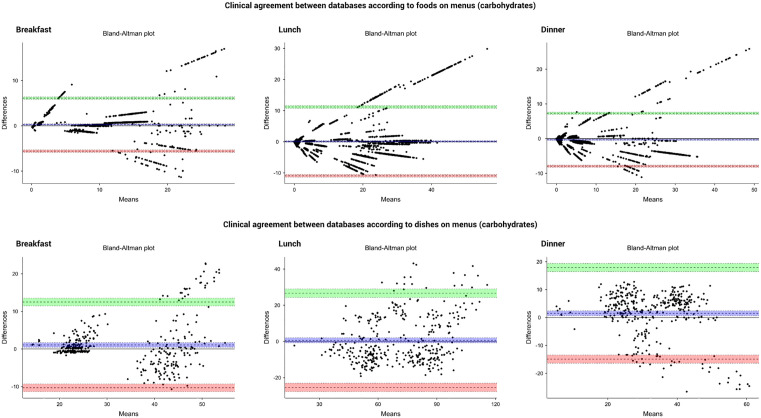
Bland-Altman plots for carbohydrate agreement between SMARTCLOTH-Database and FCDB reference models. Upper panels show agreement for individual food items; lower panels show agreement for composite dishes. Each meal type (Breakfast, Lunch, Dinner) is shown separately. Coloured lines represent mean difference (blue) and limits of agreement (green and red, ± 1.96 SD).

Finally, Fig 3 presents the Bland-Altman plots comparing the clinical agreement between the two methods for carbohydrates, as this is the most relevant immediate principle for the progression of diabetes mellitus. The three images above (1.A, 1.B, and 1.C) refer to the comparison of FOODS (breakfast, lunch and dinner, respectively); while the three lower images (2.A, 2.B, and 2.C) for DISHES (breakfast, lunch and dinner, respectively). As can be seen, the ranges between the upper and lower limits of agreement are higher for DISHES than for FOOD. In any case, the validation results are excellent.

Other Bland–Altman plots showing the level of agreement for proteins and lipids are provided in S1 and S2 Figs.

## 4. Discussion

### 4.1. Validation of SMARTCLTOH-Database

The validation of the food groups defined in the SMARTCLOTH-Database has been carried out through a comparative analysis with the FCDB database, which was designed using several reference FCDBs, considered a standard reference. Both the accuracy of nutrient estimates and the behaviour of the groups were evaluated using complete menus generated in an automated manner. Although the agreement was excellent for carbohydrates and proteins, the estimation of energy and lipids showed wider variability, particularly in complex or cooked dishes. These differences, while statistically acceptable, may have practical implications for dietary monitoring, particularly in contexts requiring precise macronutrient estimation for insulin adjustment. To address this limitation, future iterations of the SMARTCLOTH-Database will aim to refine the estimation of these nutrients through the incorporation of cooking-related adjustment factors and expanded validation in real clinical settings.

The validation results showed higher error values in complete meals than in per-food analyses, a trend widely documented in the literature. Nutritional estimation errors are more frequent in composite or cooked dishes due to the difficulty in visually breaking down ingredients, variations in recipes, and chemical modifications induced by heat treatment [14,16,28]. Amorim et al. and Meade et al. state that these factors negatively affect the accuracy of carbohydrate and other key nutrient counts in both manual and automated estimations [14,16].

The intraclass correlation coefficient (ICC) was excellent (ICC ≥ 0.75) in most cases for carbohydrate and protein, and good for lipids (ICC range 0.60-0.74) according to established interpretation criteria [25,26], indicating high agreement between the SMARTCLOTH-Database model and FCDB for the estimation of key macronutrients in diabetes management, despite slightly lower concordance for lipid estimation.

These results are consistent with those reported in previous validation studies of similar tools. For example, the study by Lopez-Sobaler et al. validated a group-structured database for population-based studies, finding over 85% agreement on energy and macronutrients using comparative analysis by food [29]. Similarly, Moreiras et al. reported deviations of less than 5% in energy, protein and fat when comparing exchange-structured FCDB against standard tables, noting that the main errors were concentrated in processed foods and mixed dishes [28].

Furthermore, the validation approach used in SMARTCLOTH, based on multiple levels (food, dish, group and daily menu) and the use of metrics such as the ICC combined with Bland and Altman charts, is consistent with the methodologies described in recent studies on the validation of technological systems for nutritional estimation [16,17]. In this regard, Abdollahzadeh et al. reported ICCs between 0.71 and 0.94 when validating the Carbulin carbohydrate counting system, results comparable to those obtained with SMARTCLOTH for meal plates (ICCs = 0.738; ICCa = 0.849 in HC) [17].

### 4.2. Implications of the use of new food groups in Diabetes Management

The incorporation of more refined food groups based on statistical nutritional criteria, such as those implemented in SMARTCLOTH-Database, represents a significant advance over traditional dietary planning schemes in patients with diabetes. Unlike models based on six broad groups [21,22] or general exchanges, the use of a detailed classification enables a more accurate estimation of macronutrients, with a smaller margin of error and improved adaptability to the patient’s nutritional profile.

This accuracy supports more accurate carbohydrate counting and personalised menu planning, which is key in the nutritional therapy of diabetes mellitus. Previous studies have shown that tools that better estimate actual macronutrient intake promote better glycaemic control, especially when integrated into strategies such as intensive carbohydrate counting [5,14].

Moreover, as the classification is adapted to the Spanish dietary pattern and differentiates between raw and cooked foods, the proposed new groups improve the practical applicability of dietary recommendations and reduce the risk of estimation errors, one of the main limitations of the classical portion method [15,28]. In this sense, the flexibility and specificity of the SMARTCLOTH-Database enable greater personalisation of the diet and better integration of local culinary preparations, which could result in improved adherence to treatment and reduced monotony in menus [7,29].

On the other hand, the computational approach based on these groups enhances the ability to generate diets automatically in a consistent and nutritionally balanced manner, which represents an opportunity for integration into intelligent clinical decision support systems, digital food education tools, and similar platforms [16,17].

Overall, the use of an advanced classification, such as the SMARTCLOTH-Database, not only provides accuracy in nutritional calculations but also opens the door to new, more individualised, culturally relevant, and technology-supported forms of dietary intervention.

### 4.3. Validation software applications for food groups according to needs or end-users

The validation software developed for this study demonstrates potential for broader applications beyond SMARTCLOTH. Unlike traditional validation approaches based on correlating nutrients with self-reported dietary data [30], the multi-scale analysis capability (by food, group, meal, and day) provides a more robust methodological approach, as demonstrated in similar work by Pozos-Parra et al. [31,32]. The modular design and use of structured food groupings enable integration with other nutrition-related projects, including automated diet generators and AI-based nutritional planning systems [33]. This approach could facilitate the development of lightweight, portable applications suitable for clinical decision support and patient-facing tools. These analytical capabilities are essential for future iterations of the model and its application in settings where nutritional accuracy has relevant clinical or epidemiological implications.

### 4.4. Limitations

Despite the positive results obtained in terms of nutrient estimation accuracy compared to FCDBs, the study has some methodological limitations that should be considered. Firstly, the validation of the SMARTCLOTH-Database was performed in a computational environment with simulated menus, based on predefined rules, which do not fully reflect the real conditions of use. The behaviour of the system has not yet been evaluated in clinical situations with real patients, where food choices and portion variability may cause unexpected errors. This limitation affects the generalisability of the results to clinical or community settings. In any case, it should be noted that the Weighing of these food groups was based on consumption data from the Spanish population, which makes it highly likely that this accurately represents the consumption of people with diabetes in Spain.

On the other hand, the model is based on group-weighted average nutritional values, which, while simplifying the calculation, may hide substantial differences between specific foods in the same group, especially those with large compositional differences. In addition, the analysis focused on macronutrients (energy, carbohydrates, protein, and lipids), excluding other key components such as fibre, sodium, vitamins, and minerals, which are also relevant in the dietary management of diabetes. Moreover, the model does not yet incorporate criteria such as sensory acceptability, individual preferences, or economic feasibility, factors that could significantly influence dietary adherence in real-life settings. Future versions of the SMARTCLOTH-Database should address these limitations by incorporating micronutrient analysis and considering user-centred factors that influence dietary adherence.

Another relevant limitation concerns the modelling of cooking-related nutrient changes. Although the SMARTCLOTH-Database differentiates between raw and cooked foods and used the Nutriplato software [24] to apply standardised retention factors, these coefficients are not yet specific to each cooking technique. Previous studies have demonstrated that nutrient losses and transformations vary considerably depending on temperature, cooking medium, and duration [28]. Integrating cooking-method-specific retention factors from established databases such as USDA [2] and FAO/INFOODS [3] would further improve the accuracy of the model in future iterations.

Furthermore, as an important direction for future research, while the present study validates the nutritional accuracy of the SMARTCLOTH-Database, it does not address user experience, usability, or acceptability of the SMARTCLOTH device itself. Currently, a clinical validation study is underway in which patients with diabetes are using the SMARTCLOTH device in their homes under real-world conditions. This ongoing research will include comprehensive usability testing and user-centred evaluation with both patients and healthcare professionals to assess practical feasibility, engagement, barriers to adoption, and perceived value of the system. Understanding these user perspectives will be essential to ensure that the technological solution effectively supports patient-centred care and long-term dietary adherence.

## 5. Conclusions

The present study has enabled the design and validation of a structured nutritional database, SMARTCLOTH-Database, by food groups, aimed at the automated calculation of energy and macronutrients in individuals with diabetes. This database, based on statistical criteria and real consumption patterns of the Spanish population, has demonstrated high agreement with an FCDB reference database, particularly in carbohydrate and protein values, key elements in the nutritional management of diabetes mellitus.

The structure, comprising 20 distinct food groups, including both raw and cooked versions, provides an excellent level of accuracy and flexibility compared to traditional classifications for these patients, which are based on only six food groups. This methodological improvement not only enables more accurate calculation of the nutritional value of menus and monitoring of dietary recommendations by the patients themselves, but also lays the groundwork for the development of advanced technological tools to support dietary education or dietary management in the home environment.

Overall, the results support the validity and applicability of SMARTCLOTH-Database as a functional core of interactive systems such as the SMARTCLOTH device. Its integration into user-centred technology solutions represents a promising advance towards more accurate, individualised and culturally tailored interventions for improving glycaemic control and dietary adherence in people with diabetes.

## Supporting information

S1 Table Food groups included in the SMARTCLOTH-Database, with corresponding nutritional composition and weighting criteria. (DOCX)

S1 Appendix Technical description of the validation software, including algorithms for menu generation and data analysis workflow. (DOCX)

S1 Data Nutritional validation dataset used for comparison between SMARTCLOTH-Database and FCDB models. (XLSX)

S1 Fig Bland–Altman plots for protein agreement between SMARTCLOTH-Database and FCDB reference models. (TIF)

S2 Fig Bland–Altman plots for lipids agreement between SMARTCLOTH-Database and FCDB reference models. (TIF)
