# Supplementary material for: Development and validation of a food group system for intake control in people with diabetes: SMARTCLOTH-Database
Source: PLOS Digit Health. 2026 Jul 9;5(7):e0001498. doi: 10.1371/journal.pdig.0001498 (PMC13349172; doi:10.1371/journal.pdig.0001498)
Supplement: S1 Table — (DOCX) [file pdig.0001498.s001.docx]

**Supplementary Table:** Full list of 199 foods included in the SMARTCLOTH-Database

| **Feeding group** | **Subgroup** | **Foods** | **Energy (Kcal)** | **Proteins (g)** | **Lipids**  **(g)** | **Carbohydrates**  **(g)** | **Source** | **Per capita consumption**  **(g)** | **Weight/group** |
| --- | --- | --- | --- | --- | --- | --- | --- | --- | --- |
| Whole milk | Raw | Curd | 92,00 | 4,80 | 5,30 | 6,70 | NUTRIPLATO | 0,21 | 0,87% |
|  | Raw | Goat milk | 65,50 | 3,40 | 3,90 | 4,50 | NUTRIPLATO | 0,18 | 0,74% |
|  | Raw | Whole UHT milk | 66,00 | 3,20 | 3,90 | 4,80 | MCYW | 19,43 | 80,29% |
|  | Raw | Fresh cheese | 105,00 | 6,80 | 7,10 | 5,70 | MCYW | 2,30 | 9,50% |
|  | Raw | Whole natural yogurt | 79,00 | 5,70 | 3,00 | 7,80 | MCYW | 2,08 | 8,60% |
| Semi-skimmed dairy | Raw | Fermented milk | 63,00 | 3,60 | 3,40 | 4,40 | BEDCA | 2,69 | 7,57% |
|  | Raw | Semi-skimmed UHT milk | 46,00 | 3,30 | 1,60 | 5,00 | MCYW | 32,85 | 92,43% |
| Skimmed milk products | Raw | Skimmed UHT milk | 34,28 | 3,30 | 0,30 | 4,90 | NUTRIPLATO | 17,71 | 88,68% |
|  | Raw | Sweetened skimmed yogurt | 31,15 | 4,00 | 0,10 | 3,80 | BEDCA | 2,26 | 11,32% |
| Sweetened dairy products | Raw | Sweetened flavoured yogurt | 93,00 | 3,20 | 3,10 | 13,20 | BEDCA | 2,04 | 53,54% |
|  | Raw | Yogurt with fruit | 78,85 | 3,10 | 1,80 | 13,40 | BEDCA | 0,44 | 11,55% |
|  | Raw | Flavoured drinking yogurt | 77,55 | 2,70 | 2,00 | 13,00 | BEDCA | 1,33 | 34,91% |
| Dairy desserts | Raw | FLAN DE VAINILLA | 99,83 | 3,00 | 1,80 | 19,10 | NUTRIPLATO | 0,97 | 19,21% |
|  | Raw | NATILLAS | 119,1 | 3,30 | 4,10 | 18,40 | NUTRIPLATO | 0,86 | 17,03% |
|  | Raw | HELADO | 228,00 | 3,80 | 11,00 | 28,20 | BEDCA | 3,22 | 63,76% |
| Sugar and sweets | Raw | White sugar | 374,25 | 0,00 | 0,00 | 99,80 | BEDCA | 3,14 | 55,87% |
|  | Raw | Sweetened cocoa powder | 363,73 | 9,80 | 8,10 | 67,10 | MCYW | 1,01 | 17,97% |
|  | Raw | Fructose | 427,00 | 11,90 | 18,70 | 52,80 | MCYW | 0,11 | 1,96% |
|  | Raw | Condensed milk | 332,50 | 8,80 | 9,70 | 56,00 | MCYW | 0,36 | 6,41% |
|  | Raw | Strawberry jam | 255,00 | 0,31 | 0,20 | 62,60 | OWN PREPARATION | 0,64 | 11,39% |
|  | Raw | Honey | 288,00 | 0,40 | 0,00 | 76,40 | MCYW | 0,36 | 6,41% |
| Vegetables | Raw | Garlic | 98,00 | 7,90 | 0,60 | 16,30 | BEDCA | 0,70 | 2,12% |
|  | Raw | Celery | 7,00 | 0,50 | 0,20 | 0,90 | MCYW | 0,42 | 1,27% |
|  | Raw | Raw onion | 31,50 | 1,50 | 0,20 | 5,30 | NUTRIPLATO | 7,12 | 21,59% |
|  | Raw | Raw white cabbage | 35,50 | 4,30 | 0,90 | 2,54 | UCM | 1,43 | 4,34% |
|  | Raw | Raw asparagus | 32,50 | 3,80 | 0,90 | 2,80 | MCYW | 0,58 | 1,76% |
|  | Raw | Lettuce | 16,65 | 1,50 | 0,60 | 1,40 | NUTRIPLATO | 4,06 | 12,31% |
|  | Raw | Raw tomato with skin | 18,12 | 1,00 | 0,35 | 3,50 | NUTRIPLATO | 12,98 | 39,36% |
|  | Raw | Raw carrot | 32,78 | 0,90 | 0,40 | 7,30 | MCYW | 3,51 | 10,64% |
|  | Raw | Raw cucumber | 12,00 | 0,70 | 0,10 | 2,00 | BEDCA | 2,18 | 6,61% |
|  | Cooked | Boiled chard | 32,01 | 1,90 | 0,58 | 4,85 | OWN PREPARATION | 0,81 | 4,57% |
|  | Cooked | Canned boiled artichoke | 17,80 | 2,90 | 0,16 | 1,20 | NUTRIPLATO | 1,21 | 6,82% |
|  | Cooked | Roasted eggplant | 58,72 | 1,27 | 4,91 | 2,51 | NUTRIPLATO | 1,68 | 9,48% |
|  | Cooked | Boiled broccoli | 24,00 | 3,10 | 0,80 | 1,10 | MCYW | 1,27 | 7,16% |
|  | Cooked | Boiled zucchini | 19,00 | 2,00 | 0,40 | 2,00 | MCYW | 4,05 | 22,84% |
|  | Cooked | Canned mushrooms | 14,00 | 0,07 | 2,25 | 0,50 | BEDCA | 1,35 | 7,61% |
|  | Cooked | Boiled cauliflower | 28,00 | 2,90 | 0,90 | 2,10 | MCYW | 0,64 | 3,61% |
|  | Cooked | Boiled green beans | 25,00 | 1,70 | 0,10 | 4,70 | MCYW | 1,67 | 9,42% |
|  | Cooked | Roasted green pepper | 14,40 | 0,76 | 0,38 | 2,49 | OWN PREPARATION | 5,05 | 28,48% |
| Fresh fruit, dried fruit and juices | Raw | Apricot | 48,40 | 0,99 | 0,11 | 11,00 | MCYW | 0,67 | 0,76% |
|  | Raw | Blueberries | 83,00 | 30,08 | 0,60 | 0,20 | BEDCA | 0,10 | 0,11% |
|  | Raw | Cherry | 48,00 | 0,90 | 0,10 | 11,50 | NUTRIPLATO | 1,25 | 1,42% |
|  | Raw | Cherimoya | 113,12 | 1,40 | 0,28 | 28,00 | NUTRIPLATO | 0,01 | 0,01% |
|  | Raw | Plum | 48,00 | 0,63 | 0,17 | 10,20 | MCYW | 1,27 | 1,44% |
|  | Raw | Raspberry | 39,00 | 0,90 | 0,60 | 8,00 | BEDCA | 0,03 | 0,03% |
|  | Raw | Strawberry | 34,45 | 0,70 | 0,60 | 7,00 | NUTRIPLATO | 2,59 | 2,94% |
|  | Raw | Kiwi | 49,00 | 1,10 | 0,50 | 10,60 | MCYW | 2,96 | 3,36% |
|  | Raw | Lemon | 19,00 | 1,00 | 0,30 | 3,20 | NUTRIPLATO | 2,31 | 2,62% |
|  | Raw | Tangerine | 35,00 | 0,90 | 0,10 | 8,00 | MCYW | 5,87 | 6,65% |
|  | Raw | Mango | 57,00 | 0,70 | 0,20 | 14,10 | MCYW | 1,08 | 1,22% |
|  | Raw | Apple | 45,00 | 0,40 | 0,10 | 11,20 | BEDCA | 9,20 | 10,43% |
|  | Raw | Peach | 72,80 | 0,84 | 0,22 | 16,80 | BEDCA | 2,50 | 2,83% |
|  | Raw | Melon | 28,00 | 0,60 | 0,10 | 6,60 | NUTRIPLATO | 7,86 | 8,91% |
|  | Raw | Orange | 44,00 | 1,10 | 0,16 | 8,90 | MCYW | 15,1 | 17,12% |
|  | Raw | Nectarine | 40,00 | 1,40 | 0,10 | 9,00 | MCYW | 1,44 | 1,63% |
|  | Raw | Pear | 41,00 | 0,30 | 0,10 | 10,40 | MCYW | 4,77 | 5,41% |
|  | Raw | Pineapple | 41,00 | 0,40 | 0,20 | 10,10 | MCYW | 2,00 | 2,27% |
|  | Raw | Banana | 85,23 | 1,20 | 0,61 | 20,80 | BEDCA | 13,54 | 15,35% |
|  | Raw | Grapefruit | 30,00 | 0,80 | 0,10 | 6,80 | MCYW | 0,29 | 0,33% |
|  | Raw | Watermelon | 31,00 | 0,50 | 0,30 | 7,10 | MCYW | 8,69 | 9,85% |
|  | Raw | White grape | 62,77 | 0,60 | 0,16 | 16,10 | NUTRIPLATO | 2,28 | 2,58% |
|  | Raw | Pineapple in syrup | 84,00 | 0,30 | 0,10 | 21,00 | NUTRIPLATO | 0,82 | 0,93% |
|  | Raw | Peach and grape juice (processed) | 49,00 | 0,60 | 0,00 | 12,00 | BEDCA | 0,45 | 0,51% |
|  | Raw | Orange juice (processed) | 41,00 | 0,60 | 0,00 | 10,00 | BEDCA | 0,17 | 0,19% |
|  | Raw | Pineapple and grape juice (processed) | 50,00 | 0,60 | 0,00 | 13,00 | BEDCA | 0,34 | 0,39% |
|  | Raw | Multifruit juice (processed) | 48,00 | 0,30 | 0,00 | 12,00 | BEDCA | 0,63 | 0,71% |
| Cereals and tubers | Cooked | Cooked white rice | 141,00 | 3,40 | 1,20 | 40,90 | OWN PREPARATION | 4,02 | 14,02% |
|  | Cooked | Cooked spaghetti | 113,00 | 4,70 | 0,90 | 23,20 | MCYW | 4,14 | 14,44% |
|  | Cooked | Boiled potato | 84,00 | 1,10 | 0,30 | 20,50 | MCYW | 20,50 | 71,55% |
|  | Raw | White rice | 364,94 | 7,54 | 1,66 | 85,28 | BEDCA | 4,00 | 6,82% |
|  | Raw | Raw spaghetti | 342,08 | 12,00 | 1,80 | 74,10 | NUTRIPLATO | 4,10 | 6,99% |
|  | Raw | White wheat bread (baguette) | 240,00 | 8,30 | 1,60 | 47,00 | BEDCA | 23,76 | 40,50% |
|  | Raw | Sliced bread | 255,43 | 5,52 | 3,74 | 53,25 | NUTRIPLATO | 6,30 | 10,75% |
|  | Raw | Raw new potato (peeled) | 78,60 | 1,90 | 0,30 | 18,80 | MCYW | 20,51 | 34,95% |
|  | Raw | Wheat flour | 348,00 | 9,30 | 1,02 | 80,00 | NUTRIPLATO | 2,49 | 2,77% |
| Legumes | Raw | Lentils | 312,80 | 23,00 | 1,70 | 54,80 | NUTRIPLATO | 1,03 | 29,15% |
|  | Raw | White beans | 304,60 | 21,40 | 1,50 | 54,80 | NUTRIPLATO | 1,00 | 28,34% |
|  | Raw | Chickpeas | 340,75 | 20,50 | 5,50 | 55,80 | NUTRIPLATO | 1,50 | 42,51% |
|  | Cooked | Cooked white beans | 116,00 | 8,80 | 0,70 | 19,90 | MCYW | 0,98 | 35,95% |
|  | Cooked | Cooked chickpeas S | 121,00 | 8,40 | 2,10 | 18,20 | MCYW | 1,48 | 54,13% |
|  | Cooked | Cooked lentils | 105,00 | 8,80 | 0,70 | 16,90 | MCYW | 0,27 | 9,92% |
| Confectionery, pastry and other | Raw | Sweetened cocoa powder | 363,73 | 9,80 | 8,10 | 67,10 | MCYW | 1,01 | 8,34% |
|  | Raw | Dark chocolate with sugar | 514,25 | 2,00 | 30,00 | 63,00 | NUTRIPLATO | 0,50 | 4,09% |
|  | Raw | Milk chocolate | 534,90 | 8,40 | 30,70 | 60,00 | NUTRIPLATO | 0,62 | 5,06% |
|  | Raw | Milk chocolate with almonds | 555,00 | 8,70 | 34,9 | 51,50 | NUTRIPLATO | 0,20 | 1,65% |
|  | Raw | Maria-type cookies | 459,75 | 7,50 | 19,00 | 69,00 | NUTRIPLATO | 5,15 | 42,41% |
|  | Raw | Milk bread | 273,00 | 6,30 | 17,50 | 24,00 | MCYW | 4,67 | 38,45% |
| Very lean protein | Raw | Prawn | 94,20 | 18,00 | 1,80 | 1,50 | NUTRIPLATO | 1,95 | 13,57% |
|  | Raw | Boiled clams and cockles | 71,20 | 15,40 | 0,40 | 1,50 | MCYW | 0,50 | 7,99% |
|  | Raw | Cooked cod fillet | 76,00 | 17,40 | 0,70 | 0,00 | MCYW | 1,20 | 19,17% |
|  | Raw | Squid | 77,00 | 16,02 | 1,10 | 0,50 | NUTRIPLATO | 1,30 | 20,77% |
|  | Raw | Sole | 77,00 | 18,00 | 1,30 | 0,30 | NUTRIPLATO | 0,20 | 3,20% |
|  | Raw | Monkfish | 70,00 | 16,70 | 0,32 | 0,40 | NUTRIPLATO | 0,30 | 4,79% |
|  | Raw | Cooked ham | 107,88 | 19,00 | 3,00 | 1,30 | NUTRIPLATO | 1,17 | 18,69% |
|  | Raw | Sea bass | 123,00 | 21,40 | 1,30 | 0,20 | NUTRIPLATO | 0,65 | 10,45% |
|  | Raw | Pickled mussels | 77,70 | 11,81 | 2,50 | 2,00 | NUTRIPLATO | 0,94 | 14,94% |
|  | Cooked | Sea bass | 123,00 | 21,40 | 1,30 | 0,20 | NUTRIPLATO | 0,70 | 11,41% |
|  | Cooked | Prawn | 107,00 | 22,60 | 1,80 | 0,00 | MCYW | 1,90 | 30,98% |
|  | Cooked | Boiled clams and cockles | 48,00 | 11,3 | 0,30 | 0,00 | MCYW | 0,49 | 7,94% |
|  | Cooked | Cooked cod fillet | 96,00 | 21,4 | 1,20 | 0,00 | NUTRIPLATO | 1,21 | 19,68% |
|  | Cooked | Grilled squid | 81,00 | 16,60 | 1,30 | 0,50 | BEDCA | 1,30 | 21,28% |
|  | Cooked | Baked sole | 72,60 | 16,00 | 0,96 | 0,00 | NUTRIPLATO | 0,22 | 3,61% |
|  | Cooked | Grilled monkfish | 93,40 | 22,00 | 0,60 | 0,00 | NUTRIPLATO | 0,31 | 5,10% |
| Lean protein foods | Raw | Gilt-head bream | 92,30 | 1,00 | 2,70 | 0,30 | BEDCA | 0,79 | 2,11% |
|  | Raw | Rabbit | 124,00 | 21,8 | 4,00 | 0,00 | MCYW | 0,80 | 6,10% |
|  | Raw | Roasted chicken thigh | 147,40 | 18,21 | 8,27 | 0,03 | NUTRIPLATO | 2,90 | 22,10% |
|  | Raw | Turbot | 108,00 | 18,30 | 3,80 | 0,30 | NUTRIPLATO | 0,10 | 0,76% |
|  | Raw | Hake | 89,00 | 15,90 | 2,80 | 0,00 | NUTRIPLATO | 2,24 | 17,10% |
|  | Raw | Veal ribs | 112,30 | 21,10 | 3,10 | 0,00 | BEDCA | 3,45 | 26,29% |
|  | Raw | Frankfurt sausage | 226,02 | 9,47 | 7,62 | 31,88 | NUTRIPLATO | 1,42 | 10,82% |
|  | Raw | Trout | 119,00 | 19,40 | 4,16 | 0,90 | NUTRIPLATO | 0,21 | 1,63% |
|  | Raw | Anchovy | 142,00 | 21,50 | 6,00 | 0,50 | NUTRIPLATO | 0,81 | 6,18% |
|  | Raw | Mussel | 72,00 | 10,18 | 1,96 | 3,40 | MCYW | 0,94 | 7,13% |
|  | Raw | Grilled white pork loin | 226,00 | 32,30 | 10,70 | 0,00 | MCYW | 0,25 | 1,90% |
|  | Cooked | Veal ribs | 112,30 | 21,10 | 3,10 | 0,00 | BEDCA | 3,50 | 15,01% |
|  | Cooked | Frankfurt sausage | 226,00 | 9,50 | 7,60 | 31,90 | NUTRIPLATO | 1,40 | 6,01% |
|  | Cooked | Trout | 119,00 | 19,40 | 4,20 | 0,90 | NUTRIPLATO | 0,20 | 0,86% |
|  | Cooked | Anchovy | 142,00 | 21,50 | 6,00 | 0,50 | NUTRIPLATO | 0,80 | 3,43% |
|  | Cooked | Mussel | 72,00 | 10,20 | 2,00 | 3,40 | MCYW | 0,90 | 3,86% |
|  | Cooked | Roasted rabbit | 121,52 | 20,71 | 3,92 | 0,00 | OWN PREPARATION | 0,8 | 3,43% |
|  | Cooked | Baked gilt-head bream | 226,00 | 32,3 | 10,7 | 0,00 | MCYW | 9,64 | 41,35% |
|  | Cooked | Roasted chicken thigh | 147,40 | 18,21 | 8,27 | 0,03 | NUTRIPLATO | 2,93 | 12,57% |
|  | Cooked | Oven-baked sea bream | 92,30 | 16,57 | 3,70 | 0,30 | NUTRIPLATO | 0,79 | 3,37% |
|  | Cooked | Baked hake | 109,16 | 6,78 | 3,01 | 5,06 | NUTRIPLATO | 2,24 | 9,63% |
|  | Cooked | Fresh turbot | 108,00 | 17,8425 | 3,80 | 0,30 | OWN PREPARATION | 0,11 | 0,47% |
| Semi-fatty protein | Cooked | Roasted lamb leg | 196,00 | 23,80 | 11,20 | 0,00 | MCYW | 0,10 | 0,07% |
|  | Cooked | Grilled beef | 132,56 | 11,90 | 12,40 | 0,00 | OWN PREPARATION | 0,10 | 0,07% |
|  | Cooked | Hen egg | 147,00 | 12,50 | 10,80 | 0,70 | MCYW | 136,00 | 91,83% |
|  | Cooked | Roasted lamb brains | 123,088 | 11,02 | 8,624 | 0,00 | OWN PREPARATION | 0,10 | 0,07% |
|  | Cooked | Turkey thigh with skin | 151,00 | 18,9 | 8,30 | 0,00 | BEDCA | 1,50 | 1,01% |
|  | Cooked | Roasted chicken thigh with skin | 147,40 | 18,21 | 8,27 | 0,03 | DIAL | 7,40 | 5,00% |
|  | Cooked | Baked salmon | 196,00 | 18,46 | 13,56 | 0,02 | NUTRIPLATO | 1,60 | 1,08% |
|  | Cooked | Sardine | 157,00 | 18,00 | 9,40 | 0,00 | NUTRIPLATO | 1,30 | 0,88% |
|  | Raw | Anchovies in oil, | 253,00 | 28,62 | 15,20 | 0,37 | NUTRIPLATO | 0,09 | 0,06% |
|  | Raw | Canned clams and cockles | 79,30 | 17,43 | 0,40 | 1,50 | BEDCA | 0,49 | 0,31% |
|  | Raw | milk-fed kid | 113,20 | 19,30 | 6,00 | 0,00 | NUTRIPLATO | 0,10 | 0,06% |
|  | Raw | Tuna in oil | 208,00 | 24,80 | 12,10 | 0,00 | NUTRIPLATO | 0,42 | 0,27% |
|  | Raw | Albacore in oil | 203,00 | 24,90 | 11,50 | 0,00 | NUTRIPLATO | 0,42 | 0,27% |
|  | Raw | Mackerel | 201,00 | 18,10 | 14,20 | 0,20 | BEDCA | 0,23 | 0,15% |
|  | Raw | Suckling kid | 113,20 | 19,30 | 6,00 | 0,00 | NUTRIPLATO | 0,08 | 0,05% |
|  | Raw | Beef | 217,00 | 16,90 | 12,10 | 0,00 | MCYW | 0,09 | 0,06% |
|  | Raw | Luncheon meat | 175,50 | 13,50 | 13,00 | 1,20 | NUTRIPLATO | 2,43 | 1,54% |
|  | Raw | Turkey luncheon meat | 131,13 | 14,50 | 7,50 | 1,50 | FEN | 0,03 | 0,02% |
|  | Raw | Chorizo | 271,88 | 27,00 | 18,00 | 0,50 | NUTRIPLATO | 0,95 | 0,60% |
|  | Raw | Hen egg | 147,00 | 12,50 | 10,80 | 0,68 | MCYW | 135,97 | 86,03% |
|  | Raw | Serrano ham | 241,38 | 31,00 | 13,00 | 0,10 | NUTRIPLATO | 1,46 | 0,92% |
|  | Raw | Lamb sweetbreads | 131,00 | 15,30 | 7,80 | 0,00 | MCYW | 0,05 | 0,03% |
|  | Raw | Turkey thigh with skin | 151,00 | 18,90 | 8,30 | 0,00 | BEDCA | 1,45 | 0,92% |
|  | Raw | Chicken thigh with skin | 230,00 | 17,60 | 17,70 | 0,00 | MCYW | 7,42 | 4,69% |
|  | Raw | Lamb leg | 186,70 | 19,00 | 12,30 | 0,00 | BEDCA | 1,00 | 0,63% |
|  | Raw | Mozzarella cheese | 233,00 | 19,90 | 16,10 | 2,20 | BEDCA | 2,3 | 1,46% |
|  | Raw | Salmon | 191,00 | 20,62 | 12,10 | 0,00 | MCYW | 1,61 | 1,02% |
|  | Raw | Smoked salmon | 140,00 | 25,40 | 14,30 | 0,00 | NUTRIPLATO | 0,18 | 0,11% |
|  | Raw | Sardine | 157,00 | 18,00 | 9,40 | 0,00 | NUTRIPLATO | 1,26 | 0,80% |
| A. PROTEICOS GRASOS  A. PROTEICOS GRASOS  A. PROTEICOS GRASOS  A. PROTEICOS GRASOS  A. PROTEICOS GRASOS  A. PROTEICOS GRASOS  A. PROTEICOS GRASOS  A. PROTEICOS GRASOS | Raw | Lamb chop | 252,50 | 17,00 | 20,50 | 0,00 | MCYW | 3,45 | 50,29% |
|  | Raw | Veal chop | 247,00 | 15,40 | 20,60 | 0,00 | NUTRIPLATO | 1,00 | 14,58% |
|  | Raw | Iberian pork | 257,00 | 18,20 | 20,50 | 0,00 | UNIVERSITY OF EXTREMADURA | 0,3 | 4,37% |
|  | Raw | Iberian ham | 374,40 | 43,20 | 22,40 | 0,00 | UNIVERSITY OF EXTREMADURA | 0,41 | 5,98% |
|  | Raw | Fresh Manchego cheese | 332,60 | 26,00 | 24,40 | 0,00 | NUTRIPLATO | 1,7 | 24,78% |
|  | Cooked | Veal chop | 252,50 | 17,00 | 20,50 | 0,00 | MCYW | 3,5 | 72,61% |
|  | Cooked | Grilled lamb chop | 355,00 | 23,50 | 29,00 | 0,00 | MCYW | 1,00 | 20,75% |
|  | Cooked | Iberian pork | 370,00 | 22,20 | 25,50 | 0,00 | UNIVERSIDAD DE EXTREMADURA | 0,32 | 6,64% |
| Very fatty protein | Raw | Foie gras | 444,00 | 10,00 | 44,00 | 3,00 | BEDCA | 0,32 | 4,28% |
|  | Raw | Cured sausage | 476,63 | 19,50 | 42,00 | 5,50 | NUTRIPLATO | 0,66 | 8,84% |
|  | Raw | Iberian loin | 318,00 | 37,50 | 18,40 | 0,60 | UNIVERSIDAD DE EXTREMADURA | 0,25 | 3,35% |
|  | Raw | Blood sausage | 429,45 | 19,50 | 37,80 | 3,00 | BEDCA | 0,06 | 0,80% |
|  | Raw | Mortadella with olives | 200,88 | 10,00 | 15,00 | 6,90 | NUTRIPLATO | 2,43 | 32,53% |
|  | Raw | Pork liver pâté | 225,00 | 10,00 | 28,00 | 2,60 | MCYW | 0,32 | 4,28% |
|  | Raw | Blue cheese | 353,50 | 21,10 | 29,60 | 0,72 | NUTRIPLATO | 0,08 | 1,07% |
|  | Raw | Cream cheese | 439,00 | 3,10 | 47,40 | 0,00 | MCYW | 0,8 | 10,71% |
|  | Raw | Cheese cured in fat | 393,00 | 9,20 | 38,22 | 3,10 | MCYW | 0,39 | 5,22% |
|  | Raw | Edam cheese | 348,50 | 29,00 | 25,00 | 2,00 | BEDCA | 0,15 | 2,01% |
|  | Raw | Emmental cheese | 380,05 | 28,00 | 29,70 | 0,20 | NUTRIPLATO | 0,1 | 1,34% |
|  | Raw | Roquefort cheese | 370,90 | 18,70 | 32,90 | 0,00 | BEDCA | 0,08 | 1,07% |
|  | Raw | Chicken sausage | 307,00 | 13,10 | 28,10 | 0,90 | BEDCA | 1,42 | 19,01% |
|  | Raw | Spanish cured sausage | 367,88 | 22,50 | 29,00 | 4,50 | NUTRIPLATO | 0,41 | 5,49% |
| Foods rich in MUFA | Raw | Sunflower oil | 882,00 | 0,00 | 99,9 | 0,00 | SENBA | 3,36 | 31,31% |
|  | Raw | Corn oil | 899,00 | 0,00 | 99,9 | 0,00 | MCYW | 0,01 | 0,09% |
|  | Raw | Olive oil | 899,10 | 0,00 | 99,9 | 0,00 | BEDCA | 0,71 | 6,62% |
|  | Raw | Extra virgin olive oil | 899,00 | 0,00 | 99,9 | 0,00 | FEN | 3,00 | 27,96% |
|  | Raw | Cottonseed oil | 899,00 | 0,00 | 99,90 | 0,00 | MCYW | 0,3 | 2,80% |
|  | Raw | Soybean oil | 899,10 | 0,00 | 99,90 | 0,00 | BEDCA | 0 | 0,00% |
|  | Raw | Avocado | 138,00 | 1,50 | 12,00 | 5,90 | FEN | 1,78 | 16,59% |
|  | Raw | Raw almond | 588,00 | 21,10 | 55,80 | 17,00 | MCYW | 0,25 | 2,33% |
|  | Raw | Hazelnut | 565,88 | 14,10 | 54,40 | 5,30 | BEDCA | 0,09 | 0,84% |
|  | Raw | Raw peanut | 637,00 | 23,00 | 49,00 | 26,00 | MCYW | 0,35 | 3,26% |
|  | Raw | Raw chestnut | 170,00 | 2,00 | 2,70 | 36,60 | MCYW | 0 | 0,00% |
|  | Raw | Walnut | 602,00 | 14,00 | 59,00 | 4,00 | NUTRIPLATO | 0,67 | 6,24% |
|  | Raw | Pistachio | 593,69 | 17,64 | 51,60 | 11,60 | NUTRIPLATO | 0,21 | 1,96% |
| SFA-rich Foods | Raw | Pork lard | 900,00 | 0,00 | 100 | 0,00 | NUTRIPLATO | 0,39 | 20,97% |
|  | Raw | Butter | 737,00 | 0,50 | 81,70 | 0,00 | MCYW | 0,4 | 21,51% |
|  | Raw | Cream | 373,00 | 2,00 | 39,30 | 3,10 | MCYW | 1,07 | 57,53% |
| PUFA-rich Foods | Raw | Sunflower oil | 882,00 | 0,00 | 99,90 | 0,00 | SENBA | 3,36 | 83,17% |
|  | Raw | Corn oil | 899,00 | 0,00 | 99,90 | 0,00 | MCYW | 0,01 | 0,25% |
|  | Raw | Soybean oil | 899,10 | 0,00 | 99,90 | 0,00 | BEDCA | 0 | 0,00% |
|  | Raw | Walnut | 602,00 | 14,00 | 59,00 | 4,00 | NUTRIPLATO | 0,67 | 16,58% |
| Other fat blends | Raw | Vegetable oi | 899,00 | 0,00 | 99,9 | 0,00 | MCYW | 3,36 | 61,31% |
|  | Raw | Margarine | 729,00 | 0,80 | 80,40 | 0,50 | BEDCA | 0,55 | 10,04% |
|  | Raw | Mayonnaise | 718,00 | 1,80 | 78,90 | 0,10 | MCYW | 1,18 | 21,53% |
|  | Raw | Pork bacon | 672,60 | 8,40 | 71,00 | 0,00 | BEDCA | 0,39 | 7,12% |
