# Supplementary material for: Development and validation of a food group system for intake control in people with diabetes: SMARTCLOTH-Database
Source: PLOS Digit Health. 2026 Jul 9;5(7):e0001498. doi: 10.1371/journal.pdig.0001498 (PMC13349172; doi:10.1371/journal.pdig.0001498)
Supplement: S1 Appendix — (DOCX) [file pdig.0001498.s002.docx]

**S1 Appendix: TECHNICAL DESCRIPTION OF THE VALIDATION SOFTWARE**

This Appendix details the implementation of the software used to validate the SMARTCLOTH model. **Fig 2** in the main article describes the general flow of the programme. The following subsections describe the structure of the files and the design of the most relevant procedures. The software is organized as a staged pipeline with a single entry point (`main.py`).

Appendix Section 1. Input file models.json

The reference file contains the structured food database used by the automatic menu generation system. Its organisation follows the logic of the **SMARTCLOTH** nutritional model, in which each food is classified according to a **food group** and, within that group, the **raw** or **cooked** subgroup. This taxonomy is part of the proposed model: all foods belonging to the same group and subgroup share the same nutritional values defined by SMARTCLOTH. This feature simplifies nutritional analysis and supports automated diet design consistent with the model.

The nutritional reference file is structured on three levels:

1. **Food group**: broad categories such as *whole dairy products*, *vegetables*, or *legumes*, which serve as the basis for the SMARTCLOTH conceptual model.
2. **Subgroup or preparation status**: allows for differentiation between raw and cooked variants relevant to intake.
3. **Individual food:** each entry contains the information necessary for its selection, nutritional characterisation, and evaluation.

Each food (level 3) is represented as an entry with associated attributes that support its nutritional characterization and its eligibility metadata within the menu generation framework, as well as the calculation of its nutritional impact under the BDCA and SMARTCLOTH reference models. Meal-compatibility attributes (Breakfast, Lunch, Dinner, Snack) are stored as food-level metadata, while effective menu composition is primarily governed by the rules defined in **generator_rules.json**. The **Appendix Table 1** describes these attributes. Nutritional values are defined per 100 grams of food.

**Appendix Table 1. Structure of models.json**

| **Attribute** | **Type** | **Description** |
| --- | --- | --- |
| Grams usual/recommended serving | float | Standard quantity in grams used by default as the unit of consumption for the food. |
| Consumption weighting | float | Value between 0 and 1 that weights the relative probability of a food being selected. |
| Breakfast | bool | True if the food can be part of a breakfast. |
| Lunch | bool | True if the food can be part of the main meal. |
| Dinner | bool | True if it can be included in a dinner. |
| Snack | bool | True if it can be used as a snack or light meal. |
| Kcal BDCA | float | Nutritional value: kilocalories per 100g, according to the BDCA database. |
| Carb BDCA | float | Nutritional value: carbohydrates per 100g (g), according to BDCA. |
| Lip BDCA | float | Nutritional value: lipids per 100g (g), according to BDCA. |
| Prot BDCA | float | Nutritional value: protein per 100g (g), according to BDCA. |
| Kcal SMARTCLOTH | float | Nutritional value: kilocalories per 100g, estimated by the SMARTCLOTH model. |
| Carb SMARTCLOTH | float | Nutritional value: carbohydrates per 100g (g), according to SMARTCLOTH. |
| Lip SMARTCLOTH | float | Nutritional value: lipids per 100g (g), according to SMARTCLOTH. |
| Prot SMARTCLOTH | float | Nutritional value: proteins per 100g (g), according to SMARTCLOTH. |

Appendix Section 2. Input file generator_rules.json

This file acts as **a specification for the menu generator**, defining the rules for combining food groups in different meals throughout a day. The information in this file configures the generation logic. It is a JSON file with hierarchical data structures. The first level of this hierarchy is described in the **Appendix Table 2**.

**Appendix Table 2. Structure of generator_rules.json**

| **Attribute** | **Type** | **Description** |
| --- | --- | --- |
| days | int | Total number of days for which menus will be generated. |
| standard_deviation_percentage | float | Standard deviation (in %) applied to quantities to introduce variability. |
| dishes | array | List of objects that define each type of meal (breakfast, lunch, etc.). |

The "dishes" attribute contains the rules for each type of meal throughout each day. For example, "breakfast" or "lunch". The structure for defining each type of dish is the next hierarchical level, described in **Appendix Table 3**.

**Appendix Table 3. Sub-structure “dish” of generator_rules.json**

| **Attribute** | **Type** | **Description** |
| --- | --- | --- |
| name | string | Name of the type of meal (e.g. "breakfast", "lunch", etc.). |
| quantity_per_day | int | Number of times a day that this meal is included. |
| menu_options | array | List of possible menu variants for this type of meal. |

Each dish may have more than one menu option, representing different combinations of food groups for its preparation. Each of these options requires a data structure described in **Appendix Table 4**, and constitutes the next hierarchical level.

**Appendix Table 4. Sub-structure “menu_option”**

| **Attribute** | **Type** | **Description** |
| --- | --- | --- |
| name | string | Menu variant identifier (e.g. "type_1", "type_2", etc.). |
| food_groups | array | Set of groups (or group alternatives) from which foods will be selected. |

The **food_groups** attribute can be defined by one or more food groups, including alternative groups from which one option is selected during menu generation. The **Appendix Table 5** describes this structure, corresponding to the last hierarchical level of information.

**Appendix Table 5. Sub-structure “food group”**

| **Attribute** | **Type** | **Description** |
| --- | --- | --- |
| group | string | Name of the food group defined in the SMARTCLOTH model. |
| raw_weighting | float (0.0 – 1.0) | (Optional) Indicates the desired proportion of raw foods in relation to cooked foods in the selection. |
| blacklist | list of strings | (Optional) List of foods to be excluded when selecting from this group. |

Appendix Section 3. Generation and analysis algorithms

**Appendix Fig 1** shows the main flow of the algorithm used to generate menus from the input files. The process begins with loading the food reference file and the generation rules. For each day and each meal type, one of the configured menu options is selected at random. Each option contains one or more food groups, which may be defined directly or as alternative groups from which one option is selected during menu generation. Within each group, blacklist exclusions are applied and, when both raw and cooked subgroups are available, the selection can be guided by a raw weighting parameter. A food item is then selected according to its consumption weighting, its portion may be adjusted by a correction factor defined at rule level and/or food level, and controlled Gaussian variability is applied to the final quantity. The result is a set of daily menus that follows the predefined structure while preserving controlled variability.





**Appendix Fig 1. Menu generation algorithm.**

**Appendix Fig 2** shows the analysis workflow used to compare nutritional values according to the BDCA and SMARTCLOTH models. The process begins with the loading of the generated menu data and the reference values of both models. Nutritional values are computed for generated menu items and are subsequently organized at the aggregation levels used in the downstream validation analysis, including food item, dish, food group, and day. The software exports spreadsheet outputs for inspection and audit, and also produces a structured validation dataset (`validation_dataset.xlsx`) that is used as the direct input for the final statistical analysis. This spreadsheet corresponds to the nutritional validation dataset supplied as Supporting Information (S3 Spreadsheet). Python performs the deterministic preparation of the analytical outputs, including nutrient computation, model differences, and dataset export, while JAMOVI is used for the final statistical evaluation of the validation dataset, including descriptive summaries, ICC(3,k), and Bland–Altman agreement analysis.

The structured validation dataset contains rows labelled according to the aggregation level used in the downstream statistical analysis, including `FOOD`, `DISH`, `GROUP`, and `DAY`, thereby allowing the direct selection of the comparison level required for each statistical procedure.

An auxiliary diagnostic step evaluates the consistency of observed food-selection frequencies against the theoretical consumption weights defined in the input food database. This check is exported as a separate spreadsheet output and is intended as a diagnostic consistency check rather than as the primary validation dataset used for the final statistical analysis.

**

**

**Appendix Fig 2. Analysis algorithm**
