# Supplementary material for: Development and validation of a food group system for intake control in people with diabetes: SMARTCLOTH-Database
Source: PLOS Digit Health. 2026 Jul 9;5(7):e0001498. doi: 10.1371/journal.pdig.0001498 (PMC13349172; doi:10.1371/journal.pdig.0001498)
Supplement: Attachment — Submitted filename: Response.pdf [file pdig.0001498.s008.pdf]

Letter to Editor and Reviewer 3

Dear Editor and Reviewer:

We would like to express our sincere gratitude for the thorough review of our manuscript and for the constructive feedback provided. We have carefully addressed all comments and suggestions, which have substantially improved the clarity, accuracy, and overall quality of the manuscript.

In this response letter, we provide a detailed point-by-point response to each comment raised during the review process. **For ease of reference, the reviewer comments are highlighted in red, and our responses to each comment are presented in blue.** Additionally, all modifications made to the manuscript are tracked using the "Track Changes" function in the revised manuscript file, allowing for clear identification of every amendment made in response to the review.

We believe that the revisions have strengthened the manuscript significantly, and we hope that the revised version now meets the standards for publication in your journal.

We remain available to address any additional questions or clarifications that may be required and would be pleased to make any further revisions deemed necessary.

Thank you once again for your valuable feedback and consideration of our manuscript.

Yours sincerely.

The authors.

RESPONSE TO REVIEWER COMMENTS

Comment 1: Abstract: Ensure the added explanation of SMARTCLOTH and comparison to existing tools is concise and does not overload the abstract. Suggest change the phrase “nutritionally coherent groups” to clearer language like “groups with similar macronutrient profiles.”

Thank you for the suggestion to make the SMARTCLOTH explanation more concise. We have streamlined the parenthetical description by removing redundancy. The revised text now reads: "SMARTCLOTH (an interactive smart tablecloth for real-time dietary monitoring in diabetes) requires a nutritionally robust and culturally adapted food database." This eliminates the repetition of "real-time dietary monitoring" that appeared twice in the original sentence, while maintaining all essential information about the device's purpose.

Thank you for this suggestion. We note that the phrase "nutritionally coherent groups" appeared in our initial submission but was already revised in the previous version of the abstract. The current terminology uses clearer language such as "groups with comparable carbohydrate, protein, and fat content" and "based on macronutrient composition similarity," which we believe addresses the reviewer's intent for more specific and accessible descriptions of the food classification system.

Comment 2: Check transitions between subsections are smooth and logical. Verify that the revised objective statement is impactful and clearly linked to the identified gap. Suggest to check terminology standardized (e.g., “dietary control” defined clearly).

Thank you for your suggestions to improve the flow and clarity of the Introduction section. We have addressed your comments as follows:

Transitions between subsections: We have added bridging sentences between subsections to ensure smooth logical progression. Specifically, transitional text has been added to connect the discussion of food composition databases with dietary monitoring strategies, and to link dietary monitoring approaches with structured classification methods. These additions help guide the reader through the rationale for developing SMARTCLOTH-Database.

Revised objective statement: We have strengthened the objective statement to make it more impactful and explicitly linked to the identified gaps. The revised objective now clearly states how SMARTCLOTH-Database addresses the limitations of current food classification systems—including estimation errors and manual data entry requirements—and emphasises that this structured approach is essential for providing users with a simplified, user-friendly dietary self-management experience through the SMARTCLOTH device.

Terminology standardisation: We have verified that key terminology is consistently used and clearly defined throughout the Introduction. The term "dietary control" is explicitly defined upon first use as "the structured and quantitative management of macronutrient intake—particularly carbohydrates—to optimise glycaemic response and medication adjustment," ensuring clarity and consistency in subsequent references.
These revisions strengthen the conceptual framework of the study and improve readability.

Comment 3: Methods: ICC model details and assumptions should be clearly stated in the main text. Confirm that the flowchart for database source selection (Figure 1) is clear and referenced properly. Ensure technical details moved to supplementary material are still accessible and well-organized.

Thank you for requesting that ICC model details and assumptions be clearly stated in the main text. We have expanded the statistical analysis section to provide comprehensive technical specifications of the ICC model. The revised text now includes: (1) the specific ICC notation (ICC(3,k) for average measures), indicating a two-way mixed-effects model; (2) explicit justification for model selection based on our study design with a fixed set of methods applied to multiple subjects; (3) clarification that ICC analyses were conducted independently at three levels of aggregation (individual food items, composite dishes, and complete daily menus); (4) explanation of why absolute agreement was chosen over consistency to

account for both systematic bias and random error; and (5) confirmation that 95% confidence intervals were calculated. These details are now fully accessible in the Methods section, ensuring transparency and reproducibility of our statistical approach.

Thank you for asking us to confirm that the flowchart for database source selection (Figure 1) is clear and properly referenced. We have verified that the figure is referenced appropriately in the Methods section where the hierarchical selection procedure is described. Regarding the clarity of the figure itself, we consider the visual representation to be clear and straightforward. However, to ensure standalone clarity and enhance reader comprehension without requiring reference to the main text, we have expanded the figure caption to provide a more detailed description. The revised caption now reads: "Figure 1. Decision flow for the selection of nutrient composition data sources. Hierarchical procedure used to select the most appropriate food composition database (BEDCA, USDA FoodData Central, or FAO/INFOODS) for each food item based on data availability, completeness, and regional representativeness." This addition ensures that readers can fully understand the figure's purpose and logic independently.

Thank you for asking us to ensure that technical details moved to supplementary material are still accessible and well-organised. We have carefully reviewed all supplementary materials and confirmed that they are properly referenced in the main text and clearly described in the Supporting Information Legends section. Specifically: (1) S1 Table (food groups with nutritional composition and weighting criteria) is referenced in the Methods section describing the database structure; (2) S2 Appendix (technical description of validation software) is referenced where the computational tool is introduced and has been updated to include section, figure, and table numbering in accordance with the journal guidelines, with all figures uploaded to the submission platform as independent supplementary files; (3) S3 Spreadsheet (nutritional validation dataset) is referenced alongside S2 Appendix; and (4) S4 and S5 Figures (Bland–Altman plots for proteins and lipids) are referenced in the Results section. Each supplementary item includes a descriptive legend that allows readers to understand its content and purpose independently. All supplementary materials remain fully accessible and well-organised to support the main manuscript.

Comment 4: Results: Verify Bland–Altman plots for all macronutrients are clearly labeled and referenced. Check consistency in decimal formatting and units (e.g., lowercase “kcal,” standardized decimal points).

Thank you for requesting that all Bland-Altman plots be clearly labelled and properly referenced. We have updated all figures to ensure comprehensive visual clarity. Each figure now includes: (1) descriptive titles specifying the macronutrient and level of analysis (foods vs. dishes); (2) clearly labelled panels identifying meal types (Breakfast, Lunch, Dinner); (3) properly labelled axes (Means, Differences); and (4) visible agreement lines colour-coded for easy interpretation (mean difference in blue, upper and lower limits of agreement in green and red). Additionally, we have enhanced the figure captions to provide standalone clarity. For example, the caption for Figure 3 (carbohydrates) now reads: "Bland-Altman plots for carbohydrate agreement between SMARTCLOTH-Database and FCDB reference models. Upper panels show agreement for individual food items; lower panels show agreement for composite dishes. Each meal type (Breakfast, Lunch, Dinner) is shown separately. Coloured lines represent mean difference (blue) and limits of agreement (green and red, ±1.96 SD)." Similar descriptive captions have been applied to S4 Figure (proteins) and S5 Figure (lipids). All figures are properly referenced in the Results section.

Thank you for requesting consistency in decimal formatting and units throughout the Results section. We have systematically reviewed and corrected all instances of unit notation and decimal formatting. Specifically: (1) we have standardised the abbreviation for kilocalories to lowercase "kcal" throughout the manuscript, including all tables (Tables 1, 2, and 3) and text, with a total of nine corrections made; and (2) we have verified and corrected decimal point formatting in Table 1, where some values used commas instead of decimal points, ensuring all numerical values now use the standard English convention with decimal points throughout the manuscript. These corrections ensure complete uniformity and adherence to standard scientific notation conventions.

Comment 5: Discussion: Confirm that statements like “clinically acceptable” are now qualified (statistically acceptable but may have practical implications). Ensure future directions (e.g., cooking-specific retention factors, usability studies) are clearly highlighted. Check that redundancy was removed and technical details are kept at a conceptual level.

Thank you for your suggestions to improve the Discussion section. We have addressed your comments as follows:

Qualification of clinical acceptability: We have expanded the statement regarding statistical acceptability to provide more context about practical implications. Additionally, we have replaced the term "adequate" with the technically correct term "good" when describing ICC values for lipids, now including the specific range (ICC 0.60-0.74) and referencing the established interpretation criteria [25,26] to ensure consistency with the Methods section.

Highlighting future directions: We have made future research directions more prominent throughout the Discussion. Specifically, we added emphasis to the paragraph on usability studies by introducing it with "as an important direction for future research," making clear that patient-centred evaluation and clinical validation in real-world settings are priority next steps. References to cooking-method-specific retention factors as areas for refinement are now clearly positioned as concrete future improvements.

Reduction of redundancy and conceptual focus: We have streamlined the Discussion to eliminate redundant content and maintain technical details at a conceptual level. Specifically: (1) we removed duplicated discussion of Nutriplato software and cooking retention factors, consolidating this content in the Limitations section where it is most appropriate; (2) we eliminated repetition of specific numerical results already presented in the Results section, retaining only the interpretative context; and (3) we substantially condensed the "Validation software applications" subsection from approximately 3,000 to 800 characters, removing implementation details whilst preserving the conceptual significance and key references [29,30,31,32].

Comment 6: Limitations: Confirm explicit mention that micronutrients (fibre, sodium, vitamins) were excluded and should be considered in future versions. Ensure usability and patient perspective are acknowledged with plans for future research.

Thank you for requesting explicit acknowledgement of excluded micronutrients and user perspective considerations. We have addressed both points in the Limitations section as follows:

Micronutrients excluded: We have expanded the discussion of excluded components to explicitly state that future versions should incorporate them. The revised text now reads: "In addition, the analysis focused on macronutrients (energy, carbohydrates, protein, and lipids), excluding other key components such as fibre, sodium, vitamins, and minerals, which are also relevant in the dietary management of diabetes. Moreover, the model does not yet incorporate criteria such as sensory acceptability, individual preferences, or economic feasibility, factors that could significantly influence dietary adherence in real-life settings. Future versions of the SMARTCLOTH-Database should address these limitations by incorporating micronutrient analysis and considering user-centred factors that influence dietary adherence."

Usability and patient perspective: We confirm that usability and patient perspectives are explicitly acknowledged with clear plans for future research. The text states: "Furthermore, as an important direction for future research, whilst the present study validates the nutritional accuracy of the SMARTCLOTH-Database, it does not address user experience, usability, or acceptability of the SMARTCLOTH device itself. Currently, a clinical validation study is underway in which patients with diabetes are using the SMARTCLOTH device in their homes under real-world conditions. This ongoing research will include comprehensive usability testing and user-centred evaluation with both patients and healthcare professionals to assess practical feasibility, engagement, barriers to adoption, and perceived value of the system." This clearly positions user-centred evaluation as a priority for ongoing research.

Comment 7: Figures & Supplementary Material: Verify that all figures have standalone clarity with proper titles and axis labels

Thank you for requesting verification that all figures have standalone clarity with proper titles and axis labels. We have comprehensively reviewed all figures and supplementary materials to ensure they meet these standards.

Main figures: All figures in the manuscript (Figures 1, 2, and 3) have been verified to include: (1) descriptive titles that clearly convey the figure's purpose and content; (2) properly labelled axes with units clearly indicated; and (3) sufficient detail in captions to allow readers to understand the figures independently of the main text. For example, Figure 3 (Bland-Altman plots) now includes a comprehensive caption describing panel organization, meal types, and the meaning of coloured lines, ensuring complete standalone clarity.

Supplementary figures: All supplementary figures (S4 and S5 Figures showing Bland-Altman plots for proteins and lipids) have been verified to maintain the same high standards of labelling and descriptive captions as the main figures, with clear axis labels, panel identifications, and comprehensive legends.

Supplementary material organization: We have verified that all supplementary materials (S1 Table, S2 Appendix, S3 Spreadsheet, S4 and S5 Figures) are properly referenced in the main text, include descriptive legends in the Supporting Information section, and are well-organised to facilitate reader access and comprehension.

All visual elements now provide complete standalone clarity whilst maintaining consistency in presentation throughout the manuscript.
