# Supplementary material for: Development and validation of a food group system for intake control in people with diabetes: SMARTCLOTH-Database
Source: PLOS Digit Health. 2026 Jul 9;5(7):e0001498. doi: 10.1371/journal.pdig.0001498 (PMC13349172; doi:10.1371/journal.pdig.0001498)
Supplement: Attachment — Submitted filename: Response_auresp_3.pdf [file pdig.0001498.s009.pdf]

Dear Editor,

We would like to thank the editorial team for the final technical recommendations provided for our manuscript. Following your guidance, we have addressed the remaining requirements concerning figure optimization and reproducibility.

In this response, we detail the actions taken to ensure the manuscript meets PLOS Digital Health's publication criteria. All modifications have been tracked in the "Revised Manuscript with Track Changes" file.

We believe these technical updates fulfill the journal's requirements and we look forward to the final decision regarding our manuscript.

Yours sincerely,

Dr. Fernando León-García On behalf of the authors.

EDITORIAL AND TECHNICAL REQUIREMENTS.

While revising your submission, we strongly recommend that you use PLOS's NAAS tool (https://ngplosjournals.pagemajik.ai/artanalysis) to test your figure files. NAAS can convert your figure files to the TIFF file type and meet basic requirements (such as print size, resolution), or provide you with a report on issues that do not meet our requirements and that NAAS cannot fix.

After uploading your figures to PLOS's NAAS tool - https://ngplosjournals.pagemajik.ai/artanalysis, NAAS will process the files provided and display the results in the "Uploaded Files" section of the page as the processing is complete. If the uploaded figures meet our requirements (or NAAS is able to fix the files to meet our requirements), the figure will be marked as "fixed" above. If NAAS is unable to fix the files, a red "failed" label will appear above. When NAAS has confirmed that the figure files meet our requirements, please download the file via the download option, and include these NAAS processed figure files when submitting your revised manuscript.

Response:

We have processed all manuscript figures through the PLOS NAAS tool. All files have been optimized and converted to the required TIFF format (300 DPI), ensuring full technical compliance with the journal's standards. These "fixed" versions are included in the current resubmission.

To enhance the reproducibility of your results, we recommend that authors of applicable studies deposit laboratory protocols in protocols.io, where a protocol can be assigned its own identifier (DOI) such that it can be cited independently in the future. Additionally, PLOS ONE offers an option to publish peer-reviewed clinical study protocols. Read more information on sharing protocols at https://plos.org/protocols?utm_medium=editorial-email&utm_source=authorletters&utm_campaign=protocols

Response:

To enhance the reproducibility of our results, we have developed a comprehensive step-by-step technical protocol and deposited it in protocols.io with the reserved DOI: 10.17504/protocols.io.x54v9q5ypl3e/v1.

This protocol is designed to provide full transparency by detailing the exact workflow for the cloning and execution of our public GitHub repository (https://github.com/i22almoj/smartcloth-gendie.git), which contains the source code for the food group generator. The guide provides a roadmap for setting up the Python-based environment and performing the subsequent data analysis using open-source statistical tools, specifically JAMOVI (version 2.3.28), ensuring that all validation results reported in our manuscript can be independently replicated without the need for proprietary software.

While the DOI is reserved and will be activated upon the manuscript's formal acceptance, the protocol remains in a private state for the editorial review process. You may access the full methodology immediately via the following private reviewer link: https://www.protocols.io/private/A5DAD42D29C311F185570A58A9FEAC02

We have also included a new section "2.5. Reproducibility and Data Availability" in the manuscript and added the protocol as a formal reference (Ref [33]).
