# Supplementary material for: Development and validation of a food group system for intake control in people with diabetes: SMARTCLOTH-Database
Source: PLOS Digit Health. 2026 Jul 9;5(7):e0001498. doi: 10.1371/journal.pdig.0001498 (PMC13349172; doi:10.1371/journal.pdig.0001498)
Supplement: Attachment — Submitted filename: Response to reviewers.docx [file pdig.0001498.s007.docx]

**Journal Requirements:**

1. Please provide separate figure files in .tif or .eps format.

For more information about figure files please see our guidelines:
[https://journals.plos.org/digitalhealth/s/figures](https://urldefense.com/v3/__https:/journals.plos.org/digitalhealth/s/figures__;!!D9dNQwwGXtA!T2J-19cPzzQj2gEjvz19ucZOvExpj94Q_1leytf-wpycOfs5PBo3OCoNIm9oyctpRGBk9Nj-0Fo111Rnox8$)
[https://journals.plos.org/digitalhealth/s/figures#loc-file-requirements](https://urldefense.com/v3/__https:/journals.plos.org/digitalhealth/s/figures*loc-file-requirements__;Iw!!D9dNQwwGXtA!T2J-19cPzzQj2gEjvz19ucZOvExpj94Q_1leytf-wpycOfs5PBo3OCoNIm9oyctpRGBk9Nj-0Fo1vG0bSfw$)

R: All figures have been uploaded in ".tif" format.

2. In the online submission form, you indicated that “The datasets and code supporting the findings of this study are available from the corresponding author upon reasonable request.”.

All PLOS journals now require all data underlying the findings described in their manuscript to be freely available to other researchers, either
a. In a public repository,
b. Within the manuscript itself, or
c. Uploaded as supplementary information.

This policy applies to all data except where public deposition would breach compliance with the protocol approved by your research ethics board. If your data cannot be made publicly available for ethical or legal reasons (e.g., public availability would compromise patient privacy), please explain your reasons by return email and your exemption request will be escalated to the editor for approval. Your exemption request will be handled independently and will not hold up the peer review process, but will need to be resolved should your manuscript be accepted for publication. One of the Editorial team will then be in touch if there are any issues.

R: In accordance with the journal's policy, the data used in this study are provided in this version of the document as supplementary material.

3. We do not publish any copyright or trademark symbols that usually accompany proprietary names, eg (R), (C), or TM (e.g. next to drug or reagent names). Please remove all instances of trademark/copyright symbols throughout the text, including “®” on page 9.

R: The registered trademark symbol has been removed.

4. If the reviewer comments include a recommendation to cite specific previously published works, please review and evaluate these publications to determine whether they are relevant and should be cited. There is no requirement to cite these works unless the editor has indicated otherwise.

-

**Reviewer #1**

This is a well-written manuscript that follows a clear structure. The technical aspects of the project (eg model creation) are detailed and well presented. There are certain areas that should be addressed to make the manuscript appropriate for publication. These areas are proposed below (minor and major recommendations).

Minor recommendations:

1. Introduction:
Paragraph 1, should FDBCAs be FCDBs?

R: Thank you for identifying this typographical error. We have corrected 'FDBCAs' to 'FCDBs' in the first paragraph of the Introduction to maintain consistency with the acronym definition provided earlier in the text (Food Composition Databases - FCDBs).

2. Methods:
Consider placing some material in a supplement section (for example some of the model tables).

R: Following the recommendations of several reviewers, five tables and two figures have been removed form this setion and moved to the supplement section.

Major recommendations:
1. Introduction:
An interesting manuscript that deviates from the norm: the main aim of the project is the development of the final product, smartcloth. However, here we are invited to evaluate only one element of the overall project, the creation of a food database. In the introduction, the authors develop a strong case about the need for smartcloth (large project) but the need for the creation and validation of a new database is not fully justified. The reader needs to search hard to find that the main reason reported for creating a new database is the inclusion of foods typical of particular cultures. This part needs more support because currently its essentiality is not convincing.

R: We sincerely appreciate this observation, which we consider very pertinent. We have revised and expanded the manuscript to clarify how nutritional modifications resulting from cooking were addressed in our study. In the Materials and Methods section (Development of the validation software), we have added an explicit description of the use of the Nutriplato software (31), developed by Moreno-Rojas et al., which applies standardised thermal retention factors to adjust the nutritional values of cooked dishes. This clarification allows the reader to understand that nutrient losses and transformations due to thermal processing were already partially considered in the study design. In the Discussion (4.1. Validation of SMARTCLOTH-Database), a new paragraph has been included acknowledging that part of the variability observed in energy and lipids may be due to differences in cooking methods. It is explained that the coefficients used by Nutriplato are general in nature and not specific to each cooking technique, which may contribute to the residual dispersion of some nutrients. Finally, in the Limitations section (4.4), a complementary paragraph has been added that addresses this issue and proposes that future versions of the SMARTCLOTH-Database integrate specific retention factors by cooking method, taking international sources such as USDA (2) and FAO/INFOODS (3) as a reference.

2. Methods
Section 2.4. The choice of ICC ranges is critical and it needs to be fully referenced.

R: Thank you for your comment. A new reference from Cicchetti (1994) has been included that supports the CCI interpretation values.

3. Results-Table 7.
This includes an important part of the analysis since it offers a first look of the performance of your model. The table description adds complexity: it states for example that the energy difference is less than 22 kcal but in the table the difference for lunch is stated as 25.7 kcal. Also, I suspect that by HC you mean CH for carbohydrate. It is an easy typo to make but since it occurs twice in three lines of text, I thought it should be checked.

R: Thank you for your comment. "HC" has been corrected to "CH" and the value of 22 to 26 Kcal.

4. Results-Figure 4
Firstly, the figure itself needs to be presented more scientifically. I appreciate that there is some explanatory text in the main body of the manuscript but graphs need to be able to stand alone and currently they all have the same title and same x and y axes. More importantly, the isolation of carbohydrates with the justification that it is the main nutrient of interest is inappropriate. Results for energy and the other macronutrients should be at least reported and not omitted entirely. Finally, the statement “In any case, the validation results are excellent” comes across as your own declaration. You need to provide the various agreement cut-offs (fully referenced), compare those against your values and then objectively report the degree of agreement. This is what you did with the ICC comparison so it is not clear why a more relaxed approach has been chosen for Bland and Altman.

R: Thank you for your comment. The clarity of Figure 4 has been improved, and new Bland-Altman plots for proteins and lipids have been created and included in the supplementary material.

5. Discussion.
I appreciate that as authors we want to emphasise the positive aspects of our research but at the same time we need to be balanced and transparent. The discussion is based on the premise that the new database is excellent and in full agreement with the gold standard. This is a generalization that is not accurate. The new database is excellent for certain aspects, for example carbohydrates and protein. However, this is not the case for energy and lipids. The choice to focus exclusively on carbohydrates (and exclude energy for example) is overly simplistic and seems convenient. The statement “The observed differences were clinically acceptable” is potentially misleading. Differences may have been statistically or mathematically acceptable but a difference of 293 kcal or 19 g of carbohydrate in one meal cannot be characterised as clinically acceptable. Single food calculations have their place in research but people consume dishes, not individual foods and this part of the analysis should have been assigned more gravity. I recommend that you accept these unfavourable results, you discuss their potential impact and suggest solutions where necessary.
Overall, I believe that this is a good manuscript and you have the opportunity to make it more transparent and comprehensive.

R: We sincerely appreciate this comment. We have revised section 4.1 (4.1. Validation of SMARTCLTOH-Database) to provide a more balanced and transparent discussion. We have added clarifications acknowledging that the deviations in estimates for carbohydrates and proteins were small, but less so for energy and lipids. The expression "clinically acceptable" was qualified, explaining that although the differences were statistically acceptable, they may have practical relevance. In addition, an explicit recommendation on the need to improve the estimation in complex dishes in future versions of the model was included in section 4.4 on limitations.

**Reviewer #2:**

The topic is important and relevant, especially as digital health tools are becoming more common in nutrition therapy. However, there are still some parts of the methods and presentation that should be improved. With some more explanations and additional data, this work can become a strong contribution to the field.

R: We sincerely thank the reviewer for their positive assessment of our work and their constructive feedback. We have carefully addressed all the specific comments raised in the detailed review, substantially revising the manuscript to improve the clarity of methods, strengthen the presentation of results, and provide additional data where requested. All specific changes are detailed below.

Major:
One important point is that nutrient values can change a lot depending on how the food is prepared—for example, when it is steamed, fried, or baked. The paper mentions that there is a difference between raw and cooked food, but it does not show how cooking changes the nutrients. It would help if the authors could add a short section in the Limitations part of the paper to explain the typical changes in nutrients due to different cooking methods. If possible, they could also use retention factors from existing tables to give an idea of the expected nutrient losses or gains.

R: We have revised and expanded the manuscript to clarify how nutritional modifications resulting from cooking were addressed in our study. In the Materials and Methods section (Development of the validation software), an explicit description of the use of Nutriplato software (24) has been added, which applies standardised heat retention factors to adjust the nutritional values of cooked dishes. This clarification enables the reader to understand that nutrient losses and transformations resulting from heat processing were already partially considered in the study design. In the Discussion (Validation of SMARTCLOTH-Database), a new paragraph has been added to acknowledge that part of the variability observed in energy and lipids may be due to differences in cooking methods. It is explained that the coefficients used by Nutriplato are general in nature and not specific to each cooking technique, which may contribute to the residual dispersion of some nutrients. Finally, a supplementary paragraph has been added to the Limitations section, addressing this issue and proposing that future versions of the SMARTCLOTH-Database integrate specific retention factors by cooking method, drawing on international sources such as the USDA (2) and FAO/INFOODS (3) as references.

Another problem is that the reader cannot know which specific foods are included in each of the 20 food groups. Since this classification is a big part of the method, it is important to include a full list of all the 199 foods and show how they are grouped. This list could be added as a supplementary table, so readers can understand how the system was built and maybe use it in their own work.

R: We appreciate your observation and consider your comment very pertinent. We have prepared a supplementary table that includes the 199 foods classified into the 20 food groups used in our study.

Also, it is not clear if the database includes branded products. For example, regular cola and diet cola can look the same but have very different sugar contents. If branded items were used, the authors should explain how they treated these items. Did they average them? Did they group them with non-branded products? One or two examples would help the reader understand this process.

R: We sincerely appreciate this observation, which allows us to clarify a critical methodological aspect. The SMARTCLOTH-Database does not include individual branded products. Instead, food consumption data published by the Spanish Ministry of Agriculture, Fisheries and Food (MAPA, 2023) [23] was used, which reflects the actual structure of national consumption by weighting the different brands and presentations of each food. In this way, the representativeness of commercial products is indirectly incorporated through the average consumption pattern. As for nutritional composition, the values come from official food composition tables (BEDCA [4], USDA [2], FAO/INFOODS [3]), which are standardised and internationally validated sources. The use of these tables instead of commercial labelling data responds to scientific criteria of homogeneity and reliability: labels may vary in formulation or analytical method, which would introduce biases in nutritional estimation. In the new version of the manuscript, a clarification has been added at the end of the Materials and Methods section (2.2. Weighing the nutritional value of the food groups and the definition of the Gold Standard for validation) to explain this methodological decision and highlight that the use of official sources ensures the consistency and comparability of the model.

Since different food composition databases sometimes give different values for the same food, the authors should describe how they chose which source to use. There is currently no clear explanation of how they decided which database value to take. A simple flow diagram or decision tree would help show the logic, and a supplementary table could list the database used for each food and the reason for that choice.

R: Indeed, different composition tables may offer slightly different values for the same food, due to methodological differences or the variability of the products analysed. To ensure the consistency of the model, we have expanded the methodological explanation in the Materials and Methods section (2.2. Weighing the nutritional value of the food groups and the definition of the Gold Standard for validation), which now details the hierarchical process of source selection. The revised text explains that the BEDCA database was the main starting point, due to its official nature and representativeness of food composition in Spain. When a food was not available in BEDCA or the information was incomplete, the USDA FoodData Central was used, and, as a last resort, the FAO/INFOODS system databases were consulted, prioritising in each case the sources that best reflected the food according to its origin and form of consumption in Spain.

It is also clarified that, when there were several options, the most recent, complete, and analytically comparable source was selected, considering the rest of the data.

In addition, a new figure (Figure 1) has been added that summarises this process using a flow chart.

In the current Limitations section, the paper only says that food processing “may” affect the nutrients. It would be better to be more specific. The authors could add a few examples or refer to published studies that show how much nutrient values usually change after cooking. This would help the reader understand if these changes are important for clinical use.

R: We appreciate your suggestion, which has greatly enriched the discussion. In Section 4.2 (Implications of Using New Food Groups in Diabetes Management), we provide a more detailed description of how cooking and processing affect nutritional content.

Minor:
There are also some small issues that should be corrected. The decimal points are written in two different ways—sometimes as commas, sometimes as periods. The same happens with the number of decimal places in tables, which are not consistent. These should be made uniform. Also, “kcal” is sometimes written with a capital “K” and sometimes with a small one. It is better to use only lowercase “kcal” to follow standard writing.

R: Thank you very much for your comment. The text and tables in the manuscript have been standardised using full stops for decimals.

In conclusion, the manuscript presents a very interesting and useful idea, and it is supported by a well-designed test. If the authors can add more explanation about how they grouped the foods, how they chose the nutrient data, and how processing was handled, the paper will be much clearer and more useful for readers and other researchers.

R: We sincerely thank the reviewer for their positive assessment of our work and their constructive feedback. We have carefully addressed all the specific comments raised, substantially revising the manuscript to improve the clarity of methods, strengthen the presentation of results, and provide additional data where requested.

**Reviewer #3:**

Thank you for the invitation to review the paper titled Development and validation of a food group system for intake control in people with diabetes: SMARTCLOTH-Database. The following review provides feedback for each section to further improve the manuscript.

R: We sincerely thank the reviewer for accepting the invitation to review our manuscript and for providing comprehensive and constructive feedback. We have carefully considered all the comments and suggestions, which have been instrumental in improving the quality of our work.

Abstract -
The acronym “SMARTCLOTH” is not explained. The abstract would benefit from a brief definition or explanation of what SMARTCLOTH stands for, especially for first-time readers. While the abstract touches on the methods used, it would benefit from slightly more specificity about the criteria for food grouping or the statistical approach to validation. This would give readers more confidence in the robustness of the system without needing to refer to the full paper. There is minimal contextualisation of how this system improves on existing databases or menu planning tools. A sentence comparing SMARTCLOTH to current alternatives (e.g. why a new grouping system was needed) would strengthen the rationale. The phrase “nutritionally coherent groups” is a bit vague—consider replacing with “groups sharing similar macronutrient profiles” or similar language for clarity.

R: We sincerely thank the reviewer for the detailed and constructive feedback on the abstract. We have carefully revised it to address all the points raised:

- SMARTCLOTH clarification: We have added a clear explanation immediately after the first mention of SMARTCLOTH, defining it as "an interactive smart tablecloth designed for dietary education and management in diabetes." This clarifies that SMARTCLOTH is not an acronym but rather the name of a wearable device, providing essential context for first-time readers.
- Increased methodological specificity: We have expanded the description of our methods to provide greater confidence in the robustness of the system. Specifically, we now explicitly state that:
- Foods were grouped based on macronutrient composition similarity (carbohydrates, proteins, and fats) and clinical utility for carbohydrate counting
- Groupings were weighted by real consumption data from Spanish national surveys
- Validation was performed using intraclass correlation coefficients (ICC)
- Multiple international databases (BEDCA, USDA, INFOODS) were used as sources
- Comparative contextualization: We have added a sentence that clearly positions SMARTCLOTH-Database relative to existing alternatives: "Unlike existing food composition databases designed primarily for epidemiological purposes, SMARTCLOTH-Database was specifically developed for automated dietary tracking, incorporating consumption-weighted data and clinically relevant food groupings tailored to diabetes management." This justifies why a new grouping system was needed and strengthens the rationale for our work.
- Terminology precision: We have replaced the vague phrase "nutritionally coherent groups" with the more specific and clearer language "groups with comparable carbohydrate, protein, and fat content," which directly describes the macronutrient similarity criterion used.

We have also applied similar improvements to the Author Summary to maintain consistency throughout the manuscript. These revisions substantially improve the clarity, specificity, and contextualization of our work without requiring readers to consult the full paper.


Introduction -
The subheadings make it very disjointed. It isn't clear early on if it is referring to Type 1 or Type 2 diabetes.

R: We appreciate your suggestion, which has greatly enriched the discussion. In Section 4.2 (Implications of Using New Food Groups in Diabetes Management), we provide a more detailed description of how cooking and processing affect nutritional content.

There are a lot of statements that are unsubstantiated and without references. What is meant by the term dietary control?
Main feedback for the introduction is -
Redundancy and Length
The introduction is overly long and at times repetitive, particularly in sections 1.2 and 1.3. For instance: Points about the benefits of carbohydrate counting and structured nutrition education are made multiple times. The psychosocial impact of dietary management, while important, could be summarised more concisely. Suggestion: Condense sections 1.2 and 1.3 by focusing on the key takeaways—clinical benefits, common tools, and limitations—without excessive elaboration or repeated citations.

R: We sincerely appreciate these comments, which have enabled us to improve the accuracy and clarity of the Introduction. After reviewing the text, we have made minor structural adjustments to improve flow and avoid repetition, without reducing essential information requested by other reviewers.

Specifically:

- It has been clarified from the first paragraph that the SMARTCLOTH model and its database have been designed to apply to both type 1 and type 2 diabetics, as both groups benefit from structured dietary management.
- The term "dietary control" has been clarified, defining it as the active planning and management of macronutrient intake, especially carbohydrates, to optimise glycaemic control and the dose of insulin or hypoglycaemic medication.
- Repetitions on carbohydrate counting and structured education have been eliminated or condensed, retaining only the primary evidence and its updated references.

We believe that these adjustments balance the various recommendations received: the Introduction maintains the conceptual depth necessary to contextualise the study, but is now written in a more direct manner that is consistent with the work's objective.

Flow and Section Integration
The transition between subsections sometimes feels abrupt. For example: The leap from dietary interventions to mobile apps (e.g., Carbulin®) is made without linking how this ties back to the need for SMARTCLOTH. The SMARTCLOTH section (1.4) reads more like a project update than a succinct framing of relevance to the current study.

Suggestion: Improve transitions between subsections with clearer linking sentences (e.g., “Given these limitations and the emergence of digital tools…”). In section 1.4, streamline the technical project timeline and focus on why SMARTCLOTH matters for the database validation in this paper.

R: We sincerely thank the reviewer for this valuable feedback. We have substantially revised the Introduction to improve transitions and streamline the SMARTCLOTH section. Specifically, we added linking sentences between subsections (e.g., "Given these limitations in traditional dietary management approaches and the growing need for more accessible and accurate tools, digital technologies have emerged as promising solutions...") to create smoother connections from dietary interventions to digital tools (Carbulin) and then to SMARTCLOTH. Each transition now explicitly addresses the limitations of previous approaches and how the next approach responds to those gaps.

Regarding Section 1.4, we have removed the detailed project timeline, technical specifications (dates, prototyping phases, design methodology details), and streamlined the content to focus exclusively on: (a) what SMARTCLOTH is and its purpose, (b) why it requires a specialized database, and (c) how the SMARTCLOTH-Database validation directly serves this need. This transforms the section from a project update into a succinct justification for the present study, as the reviewer suggested.

Objective Statement (1.5) Could Be Sharper
While the objective is presented, it lacks impact and could be more clearly linked to the gap identified earlier.

Suggestion: Consider rephrasing to something like:
“This study aims to address limitations in existing food group systems by developing and validating the SMARTCLOTH-Database—a culturally adapted, nutritionally structured food grouping system designed to support automated dietary monitoring for people with diabetes.”

R: Thank you for your comment. The objective of the study has been revised in accordance with the guidelines provided by the reviewer.

Consistency in Terminology
There's some inconsistency with terminology: "feeding," "feeding composition," and "dietary intake" are used interchangeably. “Feeding” is slightly awkward in this context and not standard in scientific English when referring to diet or food intake.

Suggestion: Replace “feeding” with “diet,” “dietary intake,” or “nutritional intake” throughout for clarity and fluency.

R: We sincerely thank the reviewer for identifying this important terminological inconsistency. We agree that the use of "feeding" is awkward and non-standard in scientific English when referring to diet or food intake. We have conducted a thorough review of the entire manuscript and systematically replaced "feeding" with more appropriate and standard terminology throughout the document. Specifically:

- "Feeding" has been replaced with "food" when referring to individual food items or food composition (e.g., "food groups," "food composition," "food selection")
- "Feeding pattern" has been replaced with "dietary pattern" when referring to eating habits
- "Feeding" has been replaced with "diet" when referring to overall dietary intake or dietary planning

These changes have been applied consistently across all sections of the manuscript, including the introduction, methods, results, discussion, tables, figures, and references. This revision substantially improves the clarity, fluency, and adherence to standard scientific terminology in nutrition research.

Methods are detailed but there are some areas that can be improved.

Terminology Consistency
The repeated use of "feeding" (e.g., feeding groups, feedingstuffs) is awkward and non-standard in scientific nutrition literature. The correct and more readable terms would be:

Food group instead of feeding group

Food item or food instead of feeding

Diet or meal pattern instead of feeding

Recommendation: Replace "feeding" terminology throughout for consistency with standard academic and clinical language.

R: We thank the reviewer for reinforcing this important point regarding terminology consistency. As detailed in our response to the previous comment on this issue, we have systematically replaced "feeding" terminology with standard scientific terms ("food," "diet," "dietary pattern") throughout the entire manuscript, including all sections, tables, figures, and references.

Complexity of Language
Some sentences are overly long and dense, particularly in the descriptions of the menu generation logic and JSON structures. While technical audiences may follow it, clarity would be improved with:

Shorter sentences

Occasional bullet points or tables

Simpler phrasing where possible

Recommendation: Break up dense blocks into more digestible sub-sections. Consider using appendices for detailed technical schema if intended for publication in a clinical or nutrition-focused journal.

R: Thank you for your comment. The subsection describing the validation software has been rewritten using shorter sentences and simplifying the technical content of the text. Furthermore, additional technical details, including the internal structure of the files and procedure diagrams, are included as supplementary material with this version.

Statistical Methods Could Be Expanded Slightly
While ICC interpretation is provided and JAMOVI is specified, the choice of ICC model (e.g., two-way random effects, single-rating, absolute agreement) is not described. Also, no mention is made of:

Assumptions testing (e.g., normality)

Sample size justification or power analysis

Recommendation: Briefly explain the ICC model used and clarify assumptions for statistical comparisons. If applicable, note how many menu simulations were generated and whether that number was sufficient for robust validation.

R: Thank you very much for your comment. A new paragraph has been added to the subsection "Validation strategy and statistical analysis", providing additional information on the aspects requested by the reviewer: a two-factor mixed-effects model with absolute agreement approach and ICC calculation based on average measures. Additionally, the methodology used for analysing data distribution has been included.

Discussion similar to the introduction there is a lot of repetition. Some statements are repeated across sections, especially around:

The advantages of refined food groups vs traditional six-group models.

The implications for better carbohydrate counting and glycaemic control.
While important, these points would be more powerful if stated once with precision.

Recommendation: Condense and streamline repeated concepts to improve flow.

R: Thank you for your insightful comment. In response, we carefully reviewed the manuscript and removed redundant sentences that repeated key concepts already addressed in other sections. These adjustments help streamline the text and improve the overall flow and conciseness of the Discussion and Conclusion sections.

Terminology Consistency
As noted in previous sections, the repeated use of "feeding," "feedingstuffs," and "feeding group" is non-standard in English-language nutrition science.

Recommendation: Replace with:

"Food group"

"Food item"

"Meal component" or simply "food"

R: We thank the reviewer for reinforcing this important point regarding terminology consistency. As detailed in our response to the previous comment on this issue, we have systematically replaced "feeding" terminology with standard scientific terms ("food," "diet," "dietary pattern") throughout the entire manuscript, including all sections, tables, figures, and references.

Technical Detail Overload in Discussion
Some of the discussion on the software structure and future applications (e.g., JSON file logic, AI feedback loops) dives too deeply into technical mechanics for a general readership in nutrition or clinical science.

Recommendation: Move technical implementation details (e.g., input file schema, algorithm steps) to supplementary material or a methods appendix, and keep the discussion at a conceptual level (e.g., how AI could use the database, not how each file structure works).

R: Thank you for your comment. The subsection describing the validation software has been rewritten to simplify the technical content of the text. Additionally, this version includes more technical details, such as the internal structure of the files and procedure diagrams, as supplementary material.

Missing Patient or Clinician Perspective
Although the SMARTCLOTH device is user-focused, the discussion lacks direct insights into user experience, usability, or preferences—even from the simulation phase. This would be valuable given the ultimate goal is patient-centred care.

Recommendation: Add at least a sentence noting future plans to assess usability, acceptability, and engagement with the tool among patients or healthcare providers.

R: We thank the reviewer for this important observation regarding the patient and clinician perspective. We agree that user experience and usability are critical components of patient-centred care. We have added a new paragraph at the end of the Limitations section acknowledging this gap and informing readers that a clinical validation study is currently underway. In this ongoing study, patients with diabetes are using the SMARTCLOTH device in their homes under real-world conditions, and comprehensive usability testing with both patients and healthcare professionals is being conducted to assess practical feasibility, engagement, and perceived value of the system. This addition addresses the reviewer's concern and provides context for future research directions.

Clarity of Language
Some phrasing is overly complex or awkward (e.g., "nutritional fidelity of the diets", "feeding selection", "dietary monotony"). These could be refined for clarity and flow.

Examples to improve:

“Feeding selection in each iteration…” → “Food selection in each menu iteration…”

“Nutritional fidelity” → “Accuracy of nutrient estimates”

R: We sincerely thank the reviewer for identifying areas where language clarity could be improved. We have conducted a thorough and meticulous review of the entire manuscript to refine overly complex or awkward phrasing. For example, we have replaced "feeding selection" with "food selection," changed "nutritional fidelity" and "nutritional concordance" to clearer terms such as "accuracy of nutrient estimates" and "agreement," simplified "dietary monotony" to "limited meal variety," and replaced "compositional heterogeneity" with "variable composition." Additionally, we have simplified unnecessarily complex phrases such as "in conjunction with" to "together with" and "adherence to nutritional treatment" to "dietary adherence." These revisions substantially improve the readability and flow of the manuscript while maintaining scientific precision and accuracy.
