# Supplementary material for: Development and validation of a food group system for intake control in people with diabetes: SMARTCLOTH-Database
Source: PLOS Digit Health. 2026 Jul 9;5(7):e0001498. doi: 10.1371/journal.pdig.0001498 (PMC13349172; doi:10.1371/journal.pdig.0001498)
Supplement: Peer review history

# Peer review: Decision Letter 0

**DOI:** 10.1371/journal.pdig.0001498.r001

**Authors:** Dukyong Yoon, Chris Easthope Awai

4 Sep 2025

PDIG-D-25-00490

Development and validation of a food group system for intake control in people with diabetes: SMARTCLOTH-Database

PLOS Digital Health

Dear Dr. León-García,

Thank you for submitting your manuscript to PLOS Digital Health. After careful consideration, we feel that it has merit but does not fully meet PLOS Digital Health's publication criteria as it currently stands. Therefore, we invite you to submit a revised version of the manuscript that addresses the points raised during the review process.

Please submit your revised manuscript within 60 days Nov 03 2025 11:59PM. If you will need more time than this to complete your revisions, please reply to this message or contact the journal office at digitalhealth@plos.org. When you're ready to submit your revision, log on to https://www.editorialmanager.com/pdig/ and select the 'Submissions Needing Revision' folder to locate your manuscript file.

Please include the following items when submitting your revised manuscript:

* A rebuttal letter that responds to each point raised by the editor and reviewer(s). You should upload this letter as a separate file labeled 'Response to Reviewers'. This file does not need to include responses to any formatting updates and technical items listed in the 'Journal Requirements' section below.

* A marked-up copy of your manuscript that highlights changes made to the original version. You should upload this as a separate file labeled 'Revised Manuscript with Track Changes'.

* An unmarked version of your revised paper without tracked changes. You should upload this as a separate file labeled 'Manuscript'.

If you would like to make changes to your financial disclosure, competing interests statement, or data availability statement, please make these updates within the submission form at the time of resubmission. Guidelines for resubmitting your figure files are available below the reviewer comments at the end of this letter.

We look forward to receiving your revised manuscript.

Kind regards,

Chris Easthope Awai, Ph.D.

Academic Editor

PLOS Digital Health

Chris Awai

Academic Editor

PLOS Digital Health

Leo Anthony Celi

Editor-in-Chief

PLOS Digital Health

orcid.org/0000-0001-6712-6626

**Journal Requirements:**

1. Please provide separate figure files in .tif or .eps format.

For more information about figure files please see our guidelines:

https://journals.plos.org/digitalhealth/s/figures

https://journals.plos.org/digitalhealth/s/figures#loc-file-requirements

2. In the online submission form, you indicated that “The datasets and code supporting the findings of this study are available from the corresponding author upon reasonable request.”.

All PLOS journals now require all data underlying the findings described in their manuscript to be freely available to other researchers, either

a. In a public repository,

b. Within the manuscript itself, or

c. Uploaded as supplementary information.

This policy applies to all data except where public deposition would breach compliance with the protocol approved by your research ethics board. If your data cannot be made publicly available for ethical or legal reasons (e.g., public availability would compromise patient privacy), please explain your reasons by return email and your exemption request will be escalated to the editor for approval. Your exemption request will be handled independently and will not hold up the peer review process, but will need to be resolved should your manuscript be accepted for publication. One of the Editorial team will then be in touch if there are any issues.

3. We do not publish any copyright or trademark symbols that usually accompany proprietary names, eg (R), (C), or TM (e.g. next to drug or reagent names). Please remove all instances of trademark/copyright symbols throughout the text, including “®” on page 9.

4. If the reviewer comments include a recommendation to cite specific previously published works, please review and evaluate these publications to determine whether they are relevant and should be cited. There is no requirement to cite these works unless the editor has indicated otherwise.

**Additional Editor Comments (if provided):**

[Note: HTML markup is below. Please do not edit.]

**Reviewers' Comments:**

Reviewer's Responses to Questions

**Comments to the Author**

1. Does this manuscript meet PLOS Digital Health’s publication criteria? Is the manuscript technically sound, and do the data support the conclusions? The manuscript must describe methodologically and ethically rigorous research with conclusions that are appropriately drawn based on the data presented.

Reviewer #1: Partly

Reviewer #2: Yes

Reviewer #3: Yes

**********

2. Has the statistical analysis been performed appropriately and rigorously?

Reviewer #1: Yes

Reviewer #2: N/A

Reviewer #3: Yes

**********

3. Have the authors made all data underlying the findings in their manuscript fully available (please refer to the Data Availability Statement at the start of the manuscript PDF file)?

The PLOS Data policy requires authors to make all data underlying the findings described in their manuscript fully available without restriction, with rare exception. The data should be provided as part of the manuscript or its supporting information, or deposited to a public repository. For example, in addition to summary statistics, the data points behind means, medians and variance measures should be available. If there are restrictions on publicly sharing data—e.g. participant privacy or use of data from a third party—those must be specified.

Reviewer #1: Yes

Reviewer #2: No

Reviewer #3: Yes

**********

4. Is the manuscript presented in an intelligible fashion and written in standard English?

PLOS Digital Health does not copyedit accepted manuscripts, so the language in submitted articles must be clear, correct, and unambiguous. Any typographical or grammatical errors should be corrected at revision, so please note any specific errors here.

Reviewer #1: Yes

Reviewer #2: Yes

Reviewer #3: No

**********

5. Review Comments to the Author

Please use the space provided to explain your answers to the questions above. You may also include additional comments for the author, including concerns about dual publication, research ethics, or publication ethics. (Please upload your review as an attachment if it exceeds 20,000 characters)

Reviewer #1: This is a well-written manuscript that follows a clear structure. The technical aspects of the project (eg model creation) are detailed and well presented. There are certain areas that should be addressed to make the manuscript appropriate for publication. These areas are proposed below (minor and major recommendations).

Minor recommendations:

1. Introduction:

Paragraph 1, should FDBCAs be FCDBs?

2. Methods:

Consider placing some material in a supplement section (for example some of the model tables).

Major recommendations:

1. Introduction:

An interesting manuscript that deviates from the norm: the main aim of the project is the development of the final product, smartcloth. However, here we are invited to evaluate only one element of the overall project, the creation of a food database. In the introduction, the authors develop a strong case about the need for smartcloth (large project) but the need for the creation and validation of a new database is not fully justified. The reader needs to search hard to find that the main reason reported for creating a new database is the inclusion of foods typical of particular cultures. This part needs more support because currently its essentiality is not convincing.

2. Methods

Section 2.4. The choice of ICC ranges is critical and it needs to be fully referenced.

3. Results-Table 7.

This includes an important part of the analysis since it offers a first look of the performance of your model. The table description adds complexity: it states for example that the energy difference is less than 22 kcal but in the table the difference for lunch is stated as 25.7 kcal. Also, I suspect that by HC you mean CH for carbohydrate. It is an easy typo to make but since it occurs twice in three lines of text, I thought it should be checked.

4. Results-Figure 4

Firstly, the figure itself needs to be presented more scientifically. I appreciate that there is some explanatory text in the main body of the manuscript but graphs need to be able to stand alone and currently they all have the same title and same x and y axes. More importantly, the isolation of carbohydrates with the justification that it is the main nutrient of interest is inappropriate. Results for energy and the other macronutrients should be at least reported and not omitted entirely. Finally, the statement “In any case, the validation results are excellent” comes across as your own declaration. You need to provide the various agreement cut-offs (fully referenced), compare those against your values and then objectively report the degree of agreement. This is what you did with the ICC comparison so it is not clear why a more relaxed approach has been chosen for Bland and Altman.

5. Discussion.

I appreciate that as authors we want to emphasise the positive aspects of our research but at the same time we need to be balanced and transparent. The discussion is based on the premise that the new database is excellent and in full agreement with the gold standard. This is a generalization that is not accurate. The new database is excellent for certain aspects, for example carbohydrates and protein. However, this is not the case for energy and lipids. The choice to focus exclusively on carbohydrates (and exclude energy for example) is overly simplistic and seems convenient. The statement “The observed differences were clinically acceptable” is potentially misleading. Differences may have been statistically or mathematically acceptable but a difference of 293 kcal or 19 g of carbohydrate in one meal cannot be characterised as clinically acceptable. Single food calculations have their place in research but people consume dishes, not individual foods and this part of the analysis should have been assigned more gravity. I recommend that you accept these unfavourable results, you discuss their potential impact and suggest solutions where necessary.

Overall, I believe that this is a good manuscript and you have the opportunity to make it more transparent and comprehensive.

Reviewer #2: The topic is important and relevant, especially as digital health tools are becoming more common in nutrition therapy. However, there are still some parts of the methods and presentation that should be improved. With some more explanations and additional data, this work can become a strong contribution to the field.

Major:

One important point is that nutrient values can change a lot depending on how the food is prepared—for example, when it is steamed, fried, or baked. The paper mentions that there is a difference between raw and cooked food, but it does not show how cooking changes the nutrients. It would help if the authors could add a short section in the Limitations part of the paper to explain the typical changes in nutrients due to different cooking methods. If possible, they could also use retention factors from existing tables to give an idea of the expected nutrient losses or gains.

Another problem is that the reader cannot know which specific foods are included in each of the 20 food groups. Since this classification is a big part of the method, it is important to include a full list of all the 199 foods and show how they are grouped. This list could be added as a supplementary table, so readers can understand how the system was built and maybe use it in their own work.

Also, it is not clear if the database includes branded products. For example, regular cola and diet cola can look the same but have very different sugar contents. If branded items were used, the authors should explain how they treated these items. Did they average them? Did they group them with non-branded products? One or two examples would help the reader understand this process.

Since different food composition databases sometimes give different values for the same food, the authors should describe how they chose which source to use. There is currently no clear explanation of how they decided which database value to take. A simple flow diagram or decision tree would help show the logic, and a supplementary table could list the database used for each food and the reason for that choice.

In the current Limitations section, the paper only says that food processing “may” affect the nutrients. It would be better to be more specific. The authors could add a few examples or refer to published studies that show how much nutrient values usually change after cooking. This would help the reader understand if these changes are important for clinical use.

Minor:

There are also some small issues that should be corrected. The decimal points are written in two different ways—sometimes as commas, sometimes as periods. The same happens with the number of decimal places in tables, which are not consistent. These should be made uniform. Also, “kcal” is sometimes written with a capital “K” and sometimes with a small one. It is better to use only lowercase “kcal” to follow standard writing.

In conclusion, the manuscript presents a very interesting and useful idea, and it is supported by a well-designed test. If the authors can add more explanation about how they grouped the foods, how they chose the nutrient data, and how processing was handled, the paper will be much clearer and more useful for readers and other researchers.

Reviewer #3: Thank you for the invitation to review the paper titled Development and validation of a food group system for intake control in people with diabetes: SMARTCLOTH-Database. The following review provides feedback for each section to further improve the manuscript.

Abstract -

The acronym “SMARTCLOTH” is not explained. The abstract would benefit from a brief definition or explanation of what SMARTCLOTH stands for, especially for first-time readers. While the abstract touches on the methods used, it would benefit from slightly more specificity about the criteria for food grouping or the statistical approach to validation. This would give readers more confidence in the robustness of the system without needing to refer to the full paper. There is minimal contextualisation of how this system improves on existing databases or menu planning tools. A sentence comparing SMARTCLOTH to current alternatives (e.g. why a new grouping system was needed) would strengthen the rationale. The phrase “nutritionally coherent groups” is a bit vague—consider replacing with “groups sharing similar macronutrient profiles” or similar language for clarity.

Introduction -

The subheadings make it very disjointed. It isn't clear early on if it is referring to Type 1 or Type 2 diabetes. There are a lot of statements that are unsubstantiated and without references. What is meant by the term dietary control?

Main feedback for the introduction is -

Redundancy and Length

The introduction is overly long and at times repetitive, particularly in sections 1.2 and 1.3. For instance: Points about the benefits of carbohydrate counting and structured nutrition education are made multiple times. The psychosocial impact of dietary management, while important, could be summarised more concisely. Suggestion: Condense sections 1.2 and 1.3 by focusing on the key takeaways—clinical benefits, common tools, and limitations—without excessive elaboration or repeated citations.

Flow and Section Integration

The transition between subsections sometimes feels abrupt. For example: The leap from dietary interventions to mobile apps (e.g., Carbulin®) is made without linking how this ties back to the need for SMARTCLOTH. The SMARTCLOTH section (1.4) reads more like a project update than a succinct framing of relevance to the current study.

Suggestion: Improve transitions between subsections with clearer linking sentences (e.g., “Given these limitations and the emergence of digital tools…”). In section 1.4, streamline the technical project timeline and focus on why SMARTCLOTH matters for the database validation in this paper.

Objective Statement (1.5) Could Be Sharper

While the objective is presented, it lacks impact and could be more clearly linked to the gap identified earlier.

Suggestion: Consider rephrasing to something like:

“This study aims to address limitations in existing food group systems by developing and validating the SMARTCLOTH-Database—a culturally adapted, nutritionally structured food grouping system designed to support automated dietary monitoring for people with diabetes.”

Consistency in Terminology

There's some inconsistency with terminology: "feeding," "feeding composition," and "dietary intake" are used interchangeably. “Feeding” is slightly awkward in this context and not standard in scientific English when referring to diet or food intake.

Suggestion: Replace “feeding” with “diet,” “dietary intake,” or “nutritional intake” throughout for clarity and fluency.

Methods are detailed but there are some areas that can be improved.

Terminology Consistency

The repeated use of "feeding" (e.g., feeding groups, feedingstuffs) is awkward and non-standard in scientific nutrition literature. The correct and more readable terms would be:

Food group instead of feeding group

Food item or food instead of feeding

Diet or meal pattern instead of feeding

Recommendation: Replace "feeding" terminology throughout for consistency with standard academic and clinical language.

Complexity of Language

Some sentences are overly long and dense, particularly in the descriptions of the menu generation logic and JSON structures. While technical audiences may follow it, clarity would be improved with:

Shorter sentences

Occasional bullet points or tables

Simpler phrasing where possible

Recommendation: Break up dense blocks into more digestible sub-sections. Consider using appendices for detailed technical schema if intended for publication in a clinical or nutrition-focused journal.

Statistical Methods Could Be Expanded Slightly

While ICC interpretation is provided and JAMOVI is specified, the choice of ICC model (e.g., two-way random effects, single-rating, absolute agreement) is not described. Also, no mention is made of:

Assumptions testing (e.g., normality)

Sample size justification or power analysis

Recommendation: Briefly explain the ICC model used and clarify assumptions for statistical comparisons. If applicable, note how many menu simulations were generated and whether that number was sufficient for robust validation.

Discussion similar to the introduction there is a lot of repetition. Some statements are repeated across sections, especially around:

The advantages of refined food groups vs traditional six-group models.

The implications for better carbohydrate counting and glycaemic control.

While important, these points would be more powerful if stated once with precision.

Recommendation: Condense and streamline repeated concepts to improve flow.

Terminology Consistency

As noted in previous sections, the repeated use of "feeding," "feedingstuffs," and "feeding group" is non-standard in English-language nutrition science.

Recommendation: Replace with:

"Food group"

"Food item"

"Meal component" or simply "food"

Technical Detail Overload in Discussion

Some of the discussion on the software structure and future applications (e.g., JSON file logic, AI feedback loops) dives too deeply into technical mechanics for a general readership in nutrition or clinical science.

Recommendation: Move technical implementation details (e.g., input file schema, algorithm steps) to supplementary material or a methods appendix, and keep the discussion at a conceptual level (e.g., how AI could use the database, not how each file structure works).

Missing Patient or Clinician Perspective

Although the SMARTCLOTH device is user-focused, the discussion lacks direct insights into user experience, usability, or preferences—even from the simulation phase. This would be valuable given the ultimate goal is patient-centred care.

Recommendation: Add at least a sentence noting future plans to assess usability, acceptability, and engagement with the tool among patients or healthcare providers.

Clarity of Language

Some phrasing is overly complex or awkward (e.g., "nutritional fidelity of the diets", "feeding selection", "dietary monotony"). These could be refined for clarity and flow.

Examples to improve:

“Feeding selection in each iteration…” → “Food selection in each menu iteration…”

“Nutritional fidelity” → “Accuracy of nutrient estimates”

**********

6. PLOS authors have the option to publish the peer review history of their article (what does this mean?). If published, this will include your full peer review and any attached files.

**Do you want your identity to be public for this peer review?**    If you choose “no”, your identity will remain anonymous but your review may still be made public.

For information about this choice, including consent withdrawal, please see our Privacy Policy.

Reviewer #1: **Yes:**   Yiannis Mavrommatis

Reviewer #2: No

Reviewer #3: No

**********

[NOTE: If reviewer comments were submitted as an attachment file, they will be attached to this email and accessible via the submission site. Please log into your account, locate the manuscript record, and check for the action link "View Attachments". If this link does not appear, there are no attachment files.]

**Figure resubmission:**

While revising your submission, we strongly recommend that you use PLOS’s NAAS tool (https://ngplosjournals.pagemajik.ai/artanalysis) to test your figure files. NAAS can convert your figure files to the TIFF file type and meet basic requirements (such as print size, resolution), or provide you with a report on issues that do not meet our requirements and that NAAS cannot fix.

After uploading your figures to PLOS’s NAAS tool - https://ngplosjournals.pagemajik.ai/artanalysis, NAAS will process the files provided and display the results in the "Uploaded Files" section of the page as the processing is complete. If the uploaded figures meet our requirements (or NAAS is able to fix the files to meet our requirements), the figure will be marked as "fixed" above. If NAAS is unable to fix the files, a red "failed" label will appear above. When NAAS has confirmed that the figure files meet our requirements, please download the file via the download option, and include these NAAS processed figure files when submitting your revised manuscript.

**Reproducibility:**

To enhance the reproducibility of your results, we recommend that authors of applicable studies deposit laboratory protocols in protocols.io, where a protocol can be assigned its own identifier (DOI) such that it can be cited independently in the future. Additionally, PLOS ONE offers an option to publish peer-reviewed clinical study protocols. Read more information on sharing protocols at https://plos.org/protocols?utm_medium=editorial-email&utm_source=authorletters&utm_campaign=protocols

# Author comment: Author response to Decision Letter 1

**DOI:** 10.1371/journal.pdig.0001498.r002

14 Nov 2025

Attachment Submitted filename: Response to reviewers.docx

# Peer review: Decision Letter 1

**DOI:** 10.1371/journal.pdig.0001498.r003

**Authors:** Dukyong Yoon, Chris Easthope Awai

18 Jan 2026

PDIG-D-25-00490R1

DEVELOPMENT AND VALIDATION OF A FOOD GROUP SYSTEM FOR INTAKE CONTROL IN PEOPLE WITH DIABETES. SMARTCLOTH-Database

PLOS Digital Health

Dear Dr. León-García,

Thank you for submitting your manuscript to PLOS Digital Health. After careful consideration, we feel that it has merit but does not fully meet PLOS Digital Health's publication criteria as it currently stands. Therefore, we invite you to submit a revised version of the manuscript that addresses the points raised during the review process.

Please submit your revised manuscript by Feb 17 2026 11:59PM. If you will need more time than this to complete your revisions, please reply to this message or contact the journal office at digitalhealth@plos.org. When you're ready to submit your revision, log on to https://www.editorialmanager.com/pdig/ and select the 'Submissions Needing Revision' folder to locate your manuscript file.

Please include the following items when submitting your revised manuscript:

* A letter that responds to each point raised by the editor and reviewer(s). You should upload this letter as a separate file labeled 'Response to Reviewers'. This file does not need to include responses to any formatting updates and technical items listed in the 'Journal Requirements' section below.

* A marked-up copy of your manuscript that highlights changes made to the original version. You should upload this as a separate file labeled 'Revised Manuscript with Track Changes'.

* An unmarked version of your revised paper without tracked changes. You should upload this as a separate file labeled 'Manuscript'.

If you would like to make changes to your financial disclosure, competing interests statement, or data availability statement, please make these updates within the submission form at the time of resubmission. Guidelines for resubmitting your figure files are available below the reviewer comments at the end of this letter.

We look forward to receiving your revised manuscript.

Kind regards,

Chris Easthope Awai

Academic Editor

PLOS Digital Health

Dukyong Yoon

Section Editor

PLOS Digital Health

Leo Anthony Celi

Editor-in-Chief

PLOS Digital Health

orcid.org/0000-0001-6712-6626

**Journal Requirements:**

If the reviewer comments include a recommendation to cite specific previously published works, please review and evaluate these publications to determine whether they are relevant and should be cited. There is no requirement to cite these works unless the editor has indicated otherwise.

**Additional Editor Comments (if provided):**

[Note: HTML markup is below. Please do not edit.]

**Reviewers' Comments:**

Reviewer's Responses to Questions

**Comments to the Author**

1. If the authors have adequately addressed your comments raised in a previous round of review and you feel that this manuscript is now acceptable for publication, you may indicate that here to bypass the “Comments to the Author” section, enter your conflict of interest statement in the “Confidential to Editor” section, and submit your "Accept" recommendation.

Reviewer #1: All comments have been addressed

Reviewer #2: All comments have been addressed

Reviewer #3: All comments have been addressed

**********

2. Does this manuscript meet PLOS Digital Health’s publication criteria? Is the manuscript technically sound, and do the data support the conclusions? The manuscript must describe methodologically and ethically rigorous research with conclusions that are appropriately drawn based on the data presented.

Reviewer #1: Yes

Reviewer #2: Yes

Reviewer #3: Yes

**********

3. Has the statistical analysis been performed appropriately and rigorously?

Reviewer #1: Yes

Reviewer #2: I don't know

Reviewer #3: Yes

**********

4. Have the authors made all data underlying the findings in their manuscript fully available (please refer to the Data Availability Statement at the start of the manuscript PDF file)?

The PLOS Data policy requires authors to make all data underlying the findings described in their manuscript fully available without restriction, with rare exception. The data should be provided as part of the manuscript or its supporting information, or deposited to a public repository. For example, in addition to summary statistics, the data points behind means, medians and variance measures should be available. If there are restrictions on publicly sharing data—e.g. participant privacy or use of data from a third party—those must be specified.

Reviewer #1: Yes

Reviewer #2: Yes

Reviewer #3: Yes

**********

5. Is the manuscript presented in an intelligible fashion and written in standard English?

PLOS Digital Health does not copyedit accepted manuscripts, so the language in submitted articles must be clear, correct, and unambiguous. Any typographical or grammatical errors should be corrected at revision, so please note any specific errors here.

Reviewer #1: Yes

Reviewer #2: Yes

Reviewer #3: Yes

**********

6. Review Comments to the Author

Please use the space provided to explain your answers to the questions above. You may also include additional comments for the author, including concerns about dual publication, research ethics, or publication ethics. (Please upload your review as an attachment if it exceeds 20,000 characters)

Reviewer #1: I would like to thank the authors for the meticulous review of the manuscript. They have covered my comments (and those from the other reviewers as much as I can judge) sufficiently.

Reviewer #2: From my perspective, all comments have been addressed in the revised version of the article. Therefore, I have no objection to its publication.

Reviewer #3: Thank you for the changes made to the original submission.

There are some minor suggestions to check/edit to further improve the manuscript.

Abstract

Ensure the added explanation of SMARTCLOTH and comparison to existing tools is concise and does not overload the abstract. Suggest change the phrase “nutritionally coherent groups” to clearer language like “groups with similar macronutrient profiles.”

Introduction

Check transitions between subsections are smooth and logical. Verify that the revised objective statement is impactful and clearly linked to the identified gap. Suggest to check terminology standardized (e.g., “dietary control” defined clearly).

Methods

ICC model details and assumptions should be clearly stated in the main text.

Confirm that the flowchart for database source selection (Figure 1) is clear and referenced properly.

Ensure technical details moved to supplementary material are still accessible and well-organized.

Results

Verify Bland–Altman plots for all macronutrients are clearly labeled and referenced.

Check consistency in decimal formatting and units (e.g., lowercase “kcal,” standardized decimal points).

Discussion

Confirm that statements like “clinically acceptable” are now qualified (statistically acceptable but may have practical implications).

Ensure future directions (e.g., cooking-specific retention factors, usability studies) are clearly highlighted.

Check that redundancy was removed and technical details are kept at a conceptual level.

Limitations

Confirm explicit mention that micronutrients (fiber, sodium, vitamins) were excluded and should be considered in future versions.

Ensure usability and patient perspective are acknowledged with plans for future research.

Figures & Supplementary Material

Verify that all figures have standalone clarity with proper titles and axis labels.

Overall: The manuscript is substantially improved and addresses major concerns. Remaining refinements relate to clarity, conciseness, and consistency.

**********

7. PLOS authors have the option to publish the peer review history of their article (what does this mean?). If published, this will include your full peer review and any attached files.

**Do you want your identity to be public for this peer review?**    If you choose “no”, your identity will remain anonymous but your review may still be made public.

For information about this choice, including consent withdrawal, please see our Privacy Policy.

Reviewer #1: **Yes:**   Yiannis Mavrommatis

Reviewer #2: No

Reviewer #3: No

**********

[NOTE: If reviewer comments were submitted as an attachment file, they will be attached to this email and accessible via the submission site. Please log into your account, locate the manuscript record, and check for the action link "View Attachments". If this link does not appear, there are no attachment files.]

**Figure resubmission:**

While revising your submission, we strongly recommend that you use PLOS’s NAAS tool (https://ngplosjournals.pagemajik.ai/artanalysis) to test your figure files. NAAS can convert your figure files to the TIFF file type and meet basic requirements (such as print size, resolution), or provide you with a report on issues that do not meet our requirements and that NAAS cannot fix.

After uploading your figures to PLOS’s NAAS tool - https://ngplosjournals.pagemajik.ai/artanalysis, NAAS will process the files provided and display the results in the "Uploaded Files" section of the page as the processing is complete. If the uploaded figures meet our requirements (or NAAS is able to fix the files to meet our requirements), the figure will be marked as "fixed" above. If NAAS is unable to fix the files, a red "failed" label will appear above. When NAAS has confirmed that the figure files meet our requirements, please download the file via the download option, and include these NAAS processed figure files when submitting your revised manuscript.

**Reproducibility:**

To enhance the reproducibility of your results, we recommend that authors of applicable studies deposit laboratory protocols in protocols.io, where a protocol can be assigned its own identifier (DOI) such that it can be cited independently in the future. Additionally, PLOS ONE offers an option to publish peer-reviewed clinical study protocols. Read more information on sharing protocols at https://plos.org/protocols?utm_medium=editorial-email&utm_source=authorletters&utm_campaign=protocols

# Author comment: Author response to Decision Letter 2

**DOI:** 10.1371/journal.pdig.0001498.r004

30 Jan 2026

Attachment Submitted filename: Response.pdf

# Editor report: Decision Letter 2

**DOI:** 10.1371/journal.pdig.0001498.r005

**Authors:** Dukyong Yoon, Chris Easthope Awai

28 Feb 2026

PDIG-D-25-00490R2

DEVELOPMENT AND VALIDATION OF A FOOD GROUP SYSTEM FOR INTAKE CONTROL IN PEOPLE WITH DIABETES. SMARTCLOTH-Database.

PLOS Digital Health

Dear Dr. León-García,

Thank you for submitting your manuscript to PLOS Digital Health. After careful consideration, we feel that it has merit but does not fully meet PLOS Digital Health's publication criteria as it currently stands. Therefore, we invite you to submit a revised version of the manuscript that addresses the points raised during the review process.

Please submit your revised manuscript by Mar 30 2026 11:59PM. If you will need more time than this to complete your revisions, please reply to this message or contact the journal office at digitalhealth@plos.org. When you're ready to submit your revision, log on to https://www.editorialmanager.com/pdig/ and select the 'Submissions Needing Revision' folder to locate your manuscript file.

Please include the following items when submitting your revised manuscript:

* A letter that responds to each point raised by the editor and reviewer(s). You should upload this letter as a separate file labeled 'Response to Reviewers'. This file does not need to include responses to any formatting updates and technical items listed in the 'Journal Requirements' section below.

* A marked-up copy of your manuscript that highlights changes made to the original version. You should upload this as a separate file labeled 'Revised Manuscript with Track Changes'.

* An unmarked version of your revised paper without tracked changes. You should upload this as a separate file labeled 'Manuscript'.

If you would like to make changes to your financial disclosure, competing interests statement, or data availability statement, please make these updates within the submission form at the time of resubmission. Guidelines for resubmitting your figure files are available below the reviewer comments at the end of this letter.

We look forward to receiving your revised manuscript.

Kind regards,

Chris Easthope Awai, Ph.D.

Academic Editor

PLOS Digital Health

Chris Awai

Academic Editor

PLOS Digital Health

Leo Anthony Celi

Editor-in-Chief

PLOS Digital Health

orcid.org/0000-0001-6712-6626

**Journal Requirements:**

1. If the reviewer comments include a recommendation to cite specific previously published works, please review and evaluate these publications to determine whether they are relevant and should be cited. There is no requirement to cite these works unless the editor has indicated otherwise.

**Additional Editor Comments (if provided):**

[Note: HTML markup is below. Please do not edit.]

**Reviewers' Comments:**

[NOTE: If reviewer comments were submitted as an attachment file, they will be attached to this email and accessible via the submission site. Please log into your account, locate the manuscript record, and check for the action link "View Attachments". If this link does not appear, there are no attachment files.]

**Figure resubmission:**

While revising your submission, we strongly recommend that you use PLOS’s NAAS tool (https://ngplosjournals.pagemajik.ai/artanalysis) to test your figure files. NAAS can convert your figure files to the TIFF file type and meet basic requirements (such as print size, resolution), or provide you with a report on issues that do not meet our requirements and that NAAS cannot fix.

After uploading your figures to PLOS’s NAAS tool - https://ngplosjournals.pagemajik.ai/artanalysis, NAAS will process the files provided and display the results in the "Uploaded Files" section of the page as the processing is complete. If the uploaded figures meet our requirements (or NAAS is able to fix the files to meet our requirements), the figure will be marked as "fixed" above. If NAAS is unable to fix the files, a red "failed" label will appear above. When NAAS has confirmed that the figure files meet our requirements, please download the file via the download option, and include these NAAS processed figure files when submitting your revised manuscript.

**Reproducibility:**

To enhance the reproducibility of your results, we recommend that authors of applicable studies deposit laboratory protocols in protocols.io, where a protocol can be assigned its own identifier (DOI) such that it can be cited independently in the future. Additionally, PLOS ONE offers an option to publish peer-reviewed clinical study protocols. Read more information on sharing protocols at https://plos.org/protocols?utm_medium=editorial-email&utm_source=authorletters&utm_campaign=protocols

# Author comment: Author response to Decision Letter 3

**DOI:** 10.1371/journal.pdig.0001498.r006

27 Mar 2026

Attachment Submitted filename: Response_auresp_3.pdf

# Editor report: Decision Letter 3

**DOI:** 10.1371/journal.pdig.0001498.r007

**Authors:** Dukyong Yoon

29 May 2026

DEVELOPMENT AND VALIDATION OF A FOOD GROUP SYSTEM FOR INTAKE CONTROL IN PEOPLE WITH DIABETES. SMARTCLOTH-Database.

PDIG-D-25-00490R3

Dear Dr. León-García,

We are pleased to inform you that your manuscript 'DEVELOPMENT AND VALIDATION OF A FOOD GROUP SYSTEM FOR INTAKE CONTROL IN PEOPLE WITH DIABETES. SMARTCLOTH-Database.' has been provisionally accepted for publication in PLOS Digital Health.

Before your manuscript can be formally accepted you will need to complete some formatting changes, which you will receive in a follow-up email from a member of our team.

Please note that your manuscript will not be scheduled for publication until you have made the required changes, so a swift response is appreciated.

IMPORTANT: The editorial review process is now complete. PLOS will only permit corrections to spelling, formatting or significant scientific errors from this point onwards. Requests for major changes, or any which affect the scientific understanding of your work, will cause delays to the publication date of your manuscript.

If your institution or institutions have a press office, please notify them about your upcoming paper to help maximize its impact. If they'll be preparing press materials, please inform our press team as soon as possible -- no later than 48 hours after receiving the formal acceptance. Your manuscript will remain under strict press embargo until 2 pm Eastern Time on the date of publication. For more information, please contact digitalhealth@plos.org.

Thank you again for supporting Open Access publishing; we are looking forward to publishing your work in PLOS Digital Health.

Best regards,

Dukyong Yoon

Section Editor

PLOS Digital Health

***********************************************************

**Additional Editor Comments (if provided):**

**Reviewer Comments (if any, and for reference):**
